# Non-solvent cinnamic acid-based gel patch for transdermal drug delivery

**DOI:** 10.1016/j.jare.2025.08.008

**Published:** 2025-08-08

**Authors:** Xi-xi Xiang, Qing-chang Xia, Xiao-bin Zhang, Yu-wei Shi, Ying-ying Yu, Pei-jie Wang, Feng-jun Ma, Min Shen, Lin-lin Zhang, Chen Chen, Meng-zhen Xing, Qing-hua Cui, Yu-ning Ma, Ting-ting Zheng, Xiao Yang

**Affiliations:** a Shandong University of Traditional Chinese Medicine, Jinan 250355, China; b The First Affiliated Hospital of Shandong First Medical University (Shandong Qianfoshan Hospital), Jinan 250014, China; c Tianjin Medical University, Tianjin 300203, China

**Keywords:** Non-solvent, Cinnamic acid, Drug-loading, Transdermal patch, Myocardial ischemia

## Abstract

**Introduction:**

Drugs entitled polymeric network special functions in bio-applications. However, the introduction of appropriate solvent for drugs generally incurred tedious inclusion methods, intensive chemical design and aging caused by solvent loss. Herein, a novel non-solvent gel system was proposed for water-insoluble drug loading and subsequent controlled transdermal drug release.

**Objectives:**

The purpose of this work is related to prepare a non-solvent transdermal gel patch loaded with cinnamic acid (CA) for the treatment of myocardial ischemia (MI), wound healing, etc.

**Methods:**

The non-solvent transdermal gel patch was easily synthesized by melting the mixture of CA, thioctic acid (TA) and zinc acetate dihydrate. Basic gel properties such as chemical architecture, mechanical strength, swelling property, rheology, adhesive property, surface hydrophilicity and antibacterial activity were studied. Biocompatibility was evaluated by *in vitro* NIH3T3 cell culture and *in vivo* subcutaneous implantation. Transdermal drug delivery was revealed by a HPLC method for *in vitro* transdermal assay. A SD rat dorsal full-thickness open wound model and a SD rat MI model were involved to verify the therapeutic effect of this gel patch.

**Results:**

The prepared gel patch contained both physical crosslinking and chemical crosslinking points. As viewed by mechanical and rheological tests, the gel exhibited ductility, viscoelasticity and self-healing property. The gel showed wide-scope adhesion towards various materials, in which the maximum tissue adhesion strength reached 0.12 MPa. The transdermal CA release rate was ranged from 20 to 40 µg/h. In prospect of histological analysis from MI treatment, this gel was also capable of repairing myocardial injury, promoting angiogenesis and inhibiting myocardial cell hypertrophy.

**Conclusion:**

This work opened an approach in the field of gel patch design for transdermal drug delivery, which was promising in large scale production and biomedical applications.

## Highlights

• Based on the concept of non-solvent transdermal patch for drug delivery, cinnamic acid was directly loaded into thioctic acid-zinc ions polymeric network. The corresponding synthetic route was facile, robust and low-cost.
• The gel patch in this research joined the merits of cinnamic acid and polymer substrate. For instance, the gel integrated multiple properties such as tissue adhesion, underwater adhesion, antibacterial, viscoelasticity, self-healing, anti-swelling, recyclability, biocompatibility, anti- inflammation, sustained drug release, among others.
• Metal acetates could also functioned as a building block for poly(thioctic acid)-based materials, resulting in a non-solvent supramolecular gel system.
• The cinnamic acid-thioctic acid-zinc ions gel could be used as a new therapy for open-wound healing, bleeding and acute myocardial ischemia.

## Graphical abstract

**Figure:**
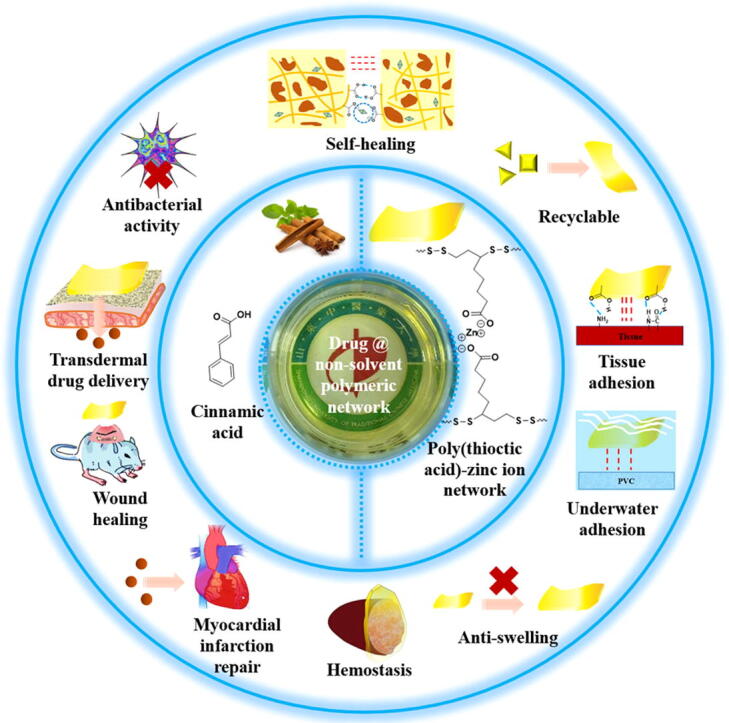

## Introduction

CA, containing a benzene ring, an alkene double bond and an acrylic acid functional group, is originated from a vast majority of natural plants including *Cinnamomum cassia* (Chinese cinnamon), *Panax ginseng*, *Liquidambar*, among others.[1] Traditional Chinese medicine reckoned that storax oil, with an abundant proportion of CA, was effective towards cardiovascular system injury such as stroke, angina pectoris and myocardial ischemia.[2,3] Nowadays, researchers have found that the medical application of CA was related to antioxidant,[4] anticancer,[5] antimicrobial[6] and antineoplastic properties.[7] Thus, the application of CA-based molecules mainly focused on therapeutic treatment of cardiovascular diseases,[8] malaria,[9] diabetes,[10] Alzheimer’s Disease,[11] other nervous system diseases,[12] exquisite pain,[13] inflammation,[14] virosis,[15] tuberculosis[16], etc. Additionally, this α,β-unsaturated carboxylic acids allowed for chemical reactions including radical addition, oxidative coupling, decarboxylation, P-mediated tandem reactions and acids related reactions,[17,18] which not only generated a bunch of CA derivatives, but also substantially expanded the corresponding potential therapeutic applications. However, the low water solubility of CA, up to 0.5 g/L, limited its medicine utilization especially in vehicle loading and controlled release. Although methods such as nanoencapsulation,[19] oil-in-water solubilization,[20] macromolecule grafting,[21] chemical modification[22] and β-cyclodextrin inclusion[23] seemed to be solutions for CA loading, intensive chemical design, time-consuming loading procedure, organic solvent dependence and environmentally unfriendly addictives were inevitable. Overall, ideal loading of CA should avoid the above limitations and should integrate CA medical properties with functional vehicles in an advanced dosage form. The properties of CA such as thermal stability, oxidation resistance and high solubility in organic phase are promising in pharmaceutical preparations without water medium.

Thioctic acid (TA) is a natural organic sulfur compound that could be polymerized to poly(TA) via heat-induced, untraviolet-induced or anionic ring-opening disulfide rearrangement.[24] Significantly, TA can form a viscous solution when the temperature exceeds 70 ^°^C, allowing the solublization of various aromatic compounds, fluorine-containing compounds, nitrile compounds, pyridine compounds and even carbon-rich nanomaterials.[25,26] As a result, the incorporation of versatile compounds entitled poly(TA) outstanding electrical, optical, self-healing properties and guaranteed the application in lithium-sulfur batteries,[27] intelligent drug-loaded nanomaterials,[28] organic photoelectric dyes[29] and other fields. Since poly(TA) contains multiple dynamic bond and inter-carboxyl hydrogen bonding, poly(TA) composites generally possessed tissue adhesion properties, in which the self-contained disulfide bonds dissipate energy to achieve extensibility and self-healing ability.[30] Thus, it could be concluded that poly(TA) was promising in drug loading as TA acted as a quasi-solvent. Ulteriorly, the tissue adhesion property of poly(TA) also enabled the application in transdermal drug delivery system (TDDS). As the best of our knowledge, the melted TA was appropriate for solubilizing drug molecules or resin compounds such as CA, paeonol and storax.

Based on the utilization of poly(TA) in TDDS, a new concept termed as non-solvent melting polymerization was proposed. The preparation process could be briefly described as “grind first and then melt”. The ingredients contained monomer, crosslinker and drug, in which one or two types of raw materials had low-melting point. Once heated above a certain temperature, polymerization accompanied by crosslinking, either chemically or physically, occurred to form a homogeneous gel system. The basic properties of non-solvent system such as swelling, rheology, tensile strength, etc. could be tuned by altering the feeding ratio of monomer/crosslinker/drug. Compared to hydrogel system, non-solvent polymerization system was not equipped with continuous aqueous phase, adaptable for long-term uses in atmosphere and water-insoluble drug loading. The drug storage function of the non-solvent system was reflected in drug aggregates phase distributed throughout the polymeric network, ensuring sustained drug delivery and controlled release. Compared to organogels such as eutectogels, both a large-scale manufacturing and a reasonable price would be achievable for this poly(TA) based non-solvent system since the corresponding preparation was efficient, pollution-free and production recyclable.[31] It could be inferred that this new dosage form could also be expanded to other drugs, other polymerization systems or other disease types. In prospect, non-solvent polymerization system effectively overcomes the limitations of liquid solvent-containing gel systems in drug loading meanwhile shows potential in large-scale production and customizable drug patches with its sustainable low-cost resources.

Herein, a simplified ingredient including CA, TA and zinc acetate dihydrate was employed to synthesize CA-based non-solvent gel (CA@TAZn). The advantages of this synthetic route could be summarized as follows: (i) The loading technique of CA was straightforward without the assistance of liposomes, amphiphilic surfactants or inclusion complex; (ii) The melted TA was capable of solubilizing metal acetate hydrate at high temperature to form a stable supramolecular network, probably caused by deacetylation under vacuum at high temperature, which strengthened the poly(TA) network as well as combined the merits of both polysulfur and metal ions; (iii) There is no need for intensive chemical design, time-consuming duration, toxic addictives or further purification processes to prepare CA@TAZn. This gel was transparent, adhesive and stretchable. As viewed by multiple chemical characterizations such as Fourier transform infrared (FTIR), X-ray photoelectron spectroscopy (XPS) and nuclear magnetic resonance (NMR), a dual-crosslinked poly(TA) network was formed accompanied by sufficient CA aggregates. The polymeric network-CA aggregates bi-phase homogenous structure was further proved by X-ray diffraction (XRD) assay, in which CA@TAZn was amorphous without post-polymerization crystallization peak. Scanning electron microscope (SEM) images demonstrated that CA@TAZn was homogeneous as well since all elements within the gel were distributed evenly. Rheology tests revealed the supramolecular semisolid nature of CA@TAZn gel patch as well as the self-healing property under step cycling strain sweep. Due to the zinc ion-based electrostatic interaction, inter-carboxyl hydrogen bonding and hydrophobic interactions, CA@TAZn gel patch demonstrated adhesion and underwater-adhesion property towards multiple materials and the adhesion strength could reach up to 0.27 MPa viewed by lap-shear tests. Furthermore, CA@TAZn also possessed tissue adhesion performance and the corresponding adhesion strength was 0.04 MPa. Other tests showed that CA@TAZn had recyclability, anti-swelling behavior, surface hydrophilicity and antibacterial property. This gel had excellent biocompatibility from the perspective of *in vitro* cellular cytotoxicity assay and *in vivo* implantation. *In vitro* transdermal tests illustrated that CA@TAZn gel patches were capable of keeping sustained CA release, in which the release rates ranged from 20 μg/h to 40 μg/h. Targeting potential clinical applications, the gel was turned out to be effective in wound healing and liver hemostasis. More importantly, the gel patch showed therapeutic effect on myocardial ischemia (MI). Overall, CA@TAZn organically combined gel features and pharmacological effects of CA, which provide a new approach for drug loading in transdermal preparations. It should be noted that the synthetic route leading to CA@TAZn gel system was inappropriate for thermally unstable active pharmaceutical ingredients. The other limitation of this work was that undesired reactions between CA and TA occurred during thermal polymerization, though the CA consuming rate was approximately 10 %.

## Experimental section

### Materials

Thioctic acid (DL *α*-lipoic acid) (AR, 99.0 %) was purchased from Aladdin Co., Shanghai, China. CA (AR, 99.5 %) was obtained from Yuanye Bio. Co., Shanghai, China. The agate mortar was produced by Heishan County Xinlitun Town Liuhe Agate Jade Factory, China. The agate mortar contained 97.26 wt% SiO_2_ and 2.74 wt% MgO/CaO/Mn_2_O_3_, with 90 mm inner diameter of and 27 mm depth. Analytically pure zinc acetate dihydrate, Zn(OAc)_2_·2H_2_O, was bought from Fuchen Chem. Co., Tianjin, China. Pigskin was purchased from Pigsland Meat Products Group Co., Ltd (Linyi, China) and stored at −20 ^°^C before uses. Phosphate buffer solution (PBS) was purchased from Sunshine Bio (Nanjing, China). Dulbecco's Modified Eagle Medium (DMEM) and pancreatin (0.25 %) was obtained from Gibco (USA). Fetal bovine serum were obtained from HyClone (USA). Acridine orange/ethidium bromide double fluorescence staining kit was obtained from Yuanye Bio. Co. Shanghai, China. HPLC grade acetonitrile was bought from ThermoFisher Scientific (USA). Nylon 66 syringe filter membranes (0.22 μm) were purchased from Jinteng Co., Tianjin, China. Ultrapure water (18 MΩ·cm resistivity) was also used in the experiments. All other reagents including methanol, ethanol, rhodamine B, dimethylsulfone (DMSO), etc. were commercial chemicals and used as received without any pretreatment.

### Preparation of CA@TAZn gels

At first, zinc acetate dihydrate was fully grinded before use. Typically, a certain amount of CA, TA and zinc acetate dihydrate were mixed and then thoroughly grinded together in a agate mortar. The detailed feeding ratios can be seen in Table 1. The mixture was added into a silicon rubber mold or a quartz mold and was subjected to 160^°^C vacuum circumstance for 1 h. A homogeneous solution was obtained afterwards, which formed a yellow transparent gel when cooled down to room temperature. Moreover, additional 1 mg rhodamine B was added to CA-TA-zinc acetate dihydrate mixture to obtain rhodamine B-stained CA@TAZn gel.

**Table 1:** Feeding ratios of CA@TAZn gels.

Sample name	Mass ratio (TA/CA/Zinc acetate dihydrate)
CA@TAZn-1	6.0:1.0:1.0
CA@TAZn-2	6.0:1.0:1.5
CA@TAZn-3	6.0:1.0:2.0
CA@TAZn-4	6.0:2.0:1.0
CA@TA	6.0: 0.1:0
TAZn	6.0:0:0.1

### Water/ethanol/PBS swelling behavior of CA@TAZn gels

CA@TAZn gel patch with a known weight were directly immersed into a given solvent (water, ethanol or PBS) at room temperature until the weight reached constant. At given points of time (10 min, 30 min, 1 h, 2 h, 3 h), CA@TAZn gel patch was removed from the given solvent and its surface was dried by filter paper, the resulted weight of gel patch was recorded (n = 3 for each kind of solvent). The swelling ratio was calculated as the following formula:

$$\mathrm{Swelling}\%\;=\;\left(\frac{m_{s}-m_{d}}{m_{d}}\right)\times 100\%$$

where *m_d_* and *m_s_* are the weight of the initial weight of CA@TAZn gel patch and the weight of the CA@TAZn gel patch after solvent soaking.

### The pH of CA@TAZn gels

CA@TAZn-1, CA@TAZn-2 and CA@TAZn-3 gel patch from three different batches (n = 3) with a weight of 0.1 g was immersed into 10 mL of newly boiled water (25°C), sonicated for 30 min to disperse evenly. Finally, the pH values were recorded by a pH meter (Mettler Toledo FE28, Switzerland & USA).

### Tensile tests

CA@TAZn gel patches (20 mm width, 20 mm length, 1 mm thickness) were used as specimens. The stress–strain curves of the gel patches were recorded by a tensile testing machine (Al-3000, GOTECH, Dongguan, China) equipped with a 500 N load cell. The tensile speed was 100 mm/min and the initial gauge length was 5 mm. The corresponding tensile strength was calculated as followed:

$$\text{Tensile strength =}\;\frac{{\text{F}}_{\text{max}}}{\text{A}}$$

where F_max_ was the maximum force and A was the cross sectional area of gel patch. Each test contained four parallel samples (n = 4). The maximum breaking elongation ratio was calculated as followed:

$$\mathrm{Maximum}\;elongation\;=\;\frac{\Delta L}{L_{0}}\times 100\%$$

where L_0_ was the initial gauge length and △L was the maximum displacement until gel broke. Moreover, the gel specimens were stretched under a cyclic load at the stretch rate of 100 mm/min with a maximum elongation of 30 mm. After 10 cycles of tensile, uniaxial tensile loading–unloading stress–strain curves were recorded at ambient conditions.

### Adhesion tests

Materials including silicon rubber (SR), aluminum (Al), glass, wood, polyvinyl chloride (PVC) and pigskin were involved in this test. Typically, the CA@TAZn gel patches (20 mm width, 20 mm length, 1 mm thickness) were fixed between 2 pieces of the given materials (25 mm width, 100 mm length) for 24 h before testing. The adhesion area was a square with a side length of 20 mm. For pigskin adhesion, the samples were sealed by plastic wrap and stored at 4 °C for 24 h before testing. For the underwater adhesion lap-shear tests, the samples were immersed in water for 24 h before testing. The stretching instrument was Al-3000, GOTECH Testing Machines, Dongguan, China. The tensile speed was 100 mm/min and each test was repeated for four times (n = 4).

### Rheology

A TA rheometer (DHR-2, USA) equipped with a 20 mm round plate was used to record the elastic modulus (G’) and the loss modulus (G”) of the CA@TAZn gel. The height of the gel during rheological test was set as 1 mm. Strain-sweep test was carried out and the strain (γ) was raised from 0.1 % to 100 %, the temperature was 25 °C and the angular frequency (ω) stayed constant at 10 rad/s. Frequency-sweep test was operated in which the ω was raised from 0.1 to 100 rad s^−1^, the temperature was 25 °C and γ stayed constant at 1 %. Moreover, a temperature-sweep test was carried out between 4 °C and 90 °C with a climbing rate of 1 °C min^−1^, in which γ and ω remained constant at 1 % and 10 rad/s, respectively. For evaluating the self-healing property of CA@TAZn gel, γ step cycling between 1 % and 150 % was conducted at 25 °C with a constant ω value of 10 rad/s.

### Contact angle test

Each water droplet with a volume of 2.0 μL was dropped onto the surface of CA@TAZn gel patches. The contact angles were recorded and measured by using a contact angle meter (KRUSS DSA25). Each test was repeated three times (n = 3).

### *In vitro* cellular cytotoxicity assay

NIH3T3 cells were obtained from ATCC. One T25 cell culture vial filled with 80–90 % NIH3T3 cells. The cells were digested with 1.5 mL trypsin for one minute, centrifuged at 4°C and 800 rpm for 5 min. Then 10 mL DMEM high glucose complete culture medium was added. 1 mL of NIH3T3 cell suspension was inoculated into each glass well and incubated for 24 h (37 °C, 5 % CO_2_) to harvest a monolayer. The remaining cell suspension was used for cell passage.

CA@TAZn gel patches were sterilized with 75 % ethyl alcohol solution and rinsed with sterilized PBS for 3 days prior to use. Gel extracts were prepared by immersing the patches in DMEM for 12 h with a concentration of 1, 5 and 10 mg/mL, respectively. The above mixture was then filtered by 0.22 μm filter membranes. The extracts were added to each glass well with NIH3T3 cell monolayer. The above cells were further incubated for another 24 h. Cells treated with DMEM were taken as the negative control while cells treated with 5 % (v/v) DMSO were taken as the positive control (n = 3).

For the detection of cell proliferation, 10 μL CCK-8 solution was added to the medium, which was then incubated for another 2 h. The adsorption intensity at 450 nm was recorded by a microplate reader (Thermo fisher, Multiskan 51119000).

For the visualization of the living/dead cells after incubation, a pipette used to aspirate the waste liquid from the culture well, then 1 mL PBS injected for cleaning and removed later. Then 1 mL acridine orange/ethidium bromide double fluorescence staining solution were added to cells removed from the culture medium. and incubate in a CO_2_ incubator for 10 min. Photos were obtained under a fluorescence microscope (SOPTOP, ICX41) at 488 nm and 520 nm wavelengths, respectively.

### HPLC method for *in vitro* transdermal assay

The concentration of CA standard solutions were 1, 15, 20, 40, 50, 100, 200 and 500 μg/mL in methanol. The HPLC integral areas of CA standard solutions were recorded by a HPLC instrument (Agilent 1260, USA) for drawing the standard curve of CA.

Porcine skin of Bama miniature pig was used as the skin model for *in vitro* transdermal assay. CA@TAZn gel patches (10 mm width, 10 mm length, 1 mm thickness) were adhered onto the Bama pigskin that fixed in a Franz diffusion cell that filled with PBS solution (pH 7.2 ± 0.2). The Franz diffusion cells were put into a transdermal diffusion device (TK-12D, Shanghai Kaikai Science and Technology Trading Co., Ltd., China) with a constant temperature of 37°C. Sample solutions at given points of time (30 min, 1 h, 2 h, 3 h, 4 h, 6 h, 8 h, 10 h, 12 h, 24 h and 48 h) were collected. All the sample solutions were sonicated for 30 min and filtered through a 0.22 μm nylon 66 syringe filter. A C18 column (4.6 mm × 250 mm, 5 μm) (Cosmosil, Japan) was used as the chromatographic column for HPLC method. Mixed solvent with a 0.3 % phosphoric acid solution /acetonitrile ratio of 65/35 (v/v) at 45°C was employed as the mobile phase. The flow rate maintained a constant value of 1.0 mL/min. The detection wavelength of CA was set as 285 nm with each injection volume of 10 μL. Each test was repeated three times (n = 3). Meanwhile, the key transdermal parameters such as transdermal flux (J_ss_) and skin permeation coefficient (K_P_) were measured according to the following equations:

$$J_{\mathrm{ss}}=\frac{\mathrm{dQ}}{\mathrm{dt}}\times \frac{1}{A}$$

$${\text{K}}_{\text{P}}=\frac{{\text{J}}_{\text{SS}}}{{\text{C}}_{\text{d}}}$$

where dQ/dt is the permeation rate; A is the effective diffusion area; C_d_ is the initial concentration of donor cell.

The *in vitro* transdermal permeation mechanism of CA@TAZn gel was evaluated through ATR-FTIR and SEM method. First, the abdominal hair of four SD rats (seven-week-old, male) were removed 24 h before the experiment. The CA@TAZn gel was applied onto the hair removal area and fixed with medical tape. After applied for 24 and 48 h, the gels were removed, respectively. The rats were euthanized and the skin was dissected. A portion of the skin tissue was fixed in 2.5 % glutaraldehyde for biological SEM analysis (ZEISS Sigma 360, GER), while another portion of the skin was dried and dehydrated at night and analyzed using the ATR-FTIR (Thermo Fisher Scientific Nicolet iS20, USA). Specifically, the skin stratum corneum side was positioned in direct contact with the diamond crystal, the angle of incidence was set at 45°, and spectra were acquired in the wavenumber range of 4000–400 cm^−1^, at 4 cm^−1^ resolution.

### Antimicrobial assay

Luria-Bertani (LB) medium was composed of tryptone (10 g), yeast extract (5 g), sodium chloride (10 g), agar (10 g) and deionized water (950 mL). The pH of the medium was adjusted to 7.0 by titration of sodium hydroxide solution (5 M). Then the volume of the LB medium was tuned to 1 L by deionized water dilution. The culture medium was further sterilized in high pressure steam pot (121°C, 0.1 MPa) for 20 min. Afterwards, the medium was placed onto the ultra-clean table and cooled down to 40°C. After gently shaking, the LB medium was poured into the bacterial culture dish and gradually solidified. The frozen *Methicillin resistant Staphylococcus aureus* (ATCC 33592) and *Escherichia coli* (ATCC 25922) strain were taken out from a −80°C freezer. The bacteria were streaked onto LB solid medium using sterile inoculation rings and were incubated at 37°C for 18 h. Bacterial clones were collected from the above culture medium. Then transferred them to a centrifuge tube containing 5 mL LB liquid culture medium. Shake the bacterial suspension in a shaker at 180 rpm and 37°C for 18 h to ensure sufficient growth and replication of bacteria in the liquid medium. The bacterial suspensions were obtained by centrifugation at 4000 rpm at room temperature for 10 min. After centrifugation, remove the supernatant and adjust the concentration of the bacterial suspension to 1 × 10^6^ CFU/mL using an appropriate amount of LB liquid culture medium.

Prior to the test, the specimens (TAZn and CA@TAZn-1 gel patches) were immersed in 75 % ethyl alcohol solution for 24 h. Then the samples were blow-dried in a super clean bench for 10 min. After sterilization and water solublization, LB medium was added into the culture dish and laid into a flat plate. Subsequently, the bacterial solution was introduced onto the medium for culturing until solidification. The previously dried samples (10 mm square, 1 mm thickness) were placed onto the culture dish. For the detection of the antibacterial activity of CA, 200 mg CA was dissolved in 4 mL ethanol solution. Then, 100 μL from such solution was dropped onto a filter papar (7 mm square) in a super clean workbench. After ethanol solution evaporated, another 4 times repeated dropping-evaporation of 100 μL CA-containing solution was carried out to make 25 mg of CA on each filter paper. The filter paper was stand in the super clean workbench for 1 h until the ethanol evaporated completely. Each resulted filter paper was placed onto the culture dish for the inhibition zone test. All the culture mediums were placed in a microbial incubator (Thermo Fisher 50125590) for 24 h. Each test contained four parallel samples (n = 4).

### Implantation of the gel patches

All animal studies were conducted in reference to the National Institutes of Health Laboratory Animal Care and Use Guidelines (NIH Publication No. 85–23 Rev. 1985) and experiments were approved by the Animal Ethics Committee of Shandong University of Traditional Chinese Medicine.

Male SD rats with an average weight of 200 g were used in this assay. The rats were randomly divided into TAZn group and CA@TAZn-1 group. After one week of adaptive feeding, the rats were anesthetized intraperitoneally with pentobarbital sodium, shaved off their back hair, disinfected with iodine, and surgically cut a wound with a length of 10 mm. Embed 10 mm square, 1 mm thickness TAZn or CA@TAZn-1 gel patch under the skin. The wound is sutured with surgical thread. After the first week, the second week, the third week, and the fourth week, respectively, the gel patch embedded on the back of the animal was taken out, weighed and photographed (n = 4 for each week). Tissue samples were taken from the vicinity of the implanted site, fixed in 10 % formalin, embedded in paraffin and sliced into 4 μm thin sections for H&E staining.

### Evaluation of hemostasis of the gel patches *in vivo*

Male Sprague-Dawley (SD) rats weighing approximately 250 g, 6–8 weeks old, were separated to four groups including blank control, positive control, TAZn and CA @TAZn-1 groups (n = 3). The TAZn and CA @TAZn-1 gel patches (20 mm width, 20 mm length, 1 mm thickness) were sterilized by ultraviolet irradiation. Sterilization on the glasswares, labwares and surgical instruments were either conducted by ethylene oxide or autoclave. Previous to the surgery, anesthesia was achieved by injecting 8 % chloral hydrate solution. In TAZn and CA @TAZn-1 gel group, once one quarter of the liver lobe was cut off, a gel patch was adhered onto the cut section area. In the positive control group, the bleeding sites were pressed with gauze instead. The bleeding site without any treatment was used as a blank control. Then a clean filter paper with a known mass (M_B_) was placed under the liver. During 3 min of bleeding, the filter paper gradually absorbed certain amount of blood and re-weighed (M_H_). The amount of blood loss was calculated by the mass variation of a filter paper before and after the blood staining: M_B_-M_H_. The hemostatic efficiency for each group was obtained by the following formula:

$$\mathrm{Hemostatic}\;efficiency\;=\;\left(M_{B}-M_{H}\right)/M_{B}\times 100\%$$

### Evaluation of skin wound healing *in vivo*

The therapeutic effect of CA@TAZn gels in wound healing was evaluated by a SD rat dorsal full-thickness open wound model. Typically, the SD rats (male, age 7–8 weeks, weight 220 ± 20 g) were raised for one week before surgery and were allowed to adapt under a desirable growth condition. All the surgical instruments undergone high-pressure sterilization. All the rats were randomly divided into four groups including blank control, PVA hydrogel (20 wt%), TAZn gel and CA@TAZn gel groups (n = 4). The rats were anesthetised via an intraperitoneal injection of sodium pentobarbital (1 wt%, 30 mg/kg). Assisted by dissection scissors, a loop cutter was utilized to make a complete-thickness skin wound with 10 mm diameter on each rat back. The wounds of the blank control group undergone non-treatment. Meanwhile, the wounds in other groups were covered with the PVA hydrogel, TAZn gel and CA@TAZn gel (20 mm square, 1 mm thickness), respectively. Afterwards, all the groups were covered with a sterile gauze fixed onto the back of the rats with a bandage. The conditions of wounds were photographed with a digital camera at certain time points (day 0, day 1, day 3, day 5, day 7, day 10 and day 14), and the wound area was recorded according to ImageJ software. The wound healing rate was calculated as follows:

$$\mathrm{Wound}\;healing\;rate\;=\;\frac{S_{0}-S_{n}}{S_{0}}\times 100\%$$

where S0–S14 are the wound areas on day 0–14, respectively. On the 14th day, the wound tissues were post-fixed in a 4 % paraformaldehyde solution, and then dehydrated sequentially with 50 %, 70 %, 80 %, 90 %, 95 %, and 100 % ethanol solutions for 30 min. The wound tissues were embedded in paraffin and then sliced in approximately 5 μm thick pieces for H&E and Masson staining analysis. ImageJ software was employed to analyze the healing thickness of the wound and the collagen content within the tissue.

### Cardiac function evaluation in the treatment of MI model

Healthy male SD rats aging approximately 8 weeks were randomly divided into sham group, MI group, TAZn group and CA@TAZn-1 group (n = 4). All the rats were anesthetized with urethane (1 g/kg) assisted by mechanical ventilation. The ligation operation of the left anterior descending artery (LAD) was operated after a left lateral thoracotomy and pericardectomy. Both the width and depth of ligation were 2 mm. Phenomena such as significant ST segment elevation in the electrocardiogram as well as white or purple left apex area indicated successful ligation. In the sham group, only thoracotomy was conducted without LAD. In the TAZn group and CA@TAZn-1 group, gel patches (10 mm square, 1 mm thickness) were adhered onto BL15 (Xinshu) acupuncturing points with a 48 h adhesion duration of each gel patch. The treatment period was 6 days after ligation.

For histological analysis, the hearts of all rats were obtained after the treatment period and cut into frozen sections with a 6 μm thickness. Masson tricolor staining was employed to view the infarcted area of heart according to the ratio of collagenous section (blue) to myocardial section (red). The calculation was done by Image J and Originlab software. Moreover, immunofluorescence staining was also carried out. Typically, the cardiac sections were rinsed 3 times in PBS and permeabilized with 0.2 % Triton X-100 at 25°C for 10 min. Subsequently, the cardiac sections were washed by PBS again and then blocked in 5 % BSA (1 g BSA in 20 mL PBS) for 30 min for. After gently removed block solution, the primary antibodies of WGA (1:200), vWF (1:250) and α-SMA (1:200) were added, then stored at 4 °C overnight. After PBS washing, secondary antibodies work solution was added to the samples for 30 min. At last, the nucleuses were labeled with DAPI. The microimages were obtained using fluorescence microscope (Nikon, Ts-2R).

### Other characterizations

Infrared spectra were characterized by a FT-IR spectrometer (Frontier, USA). Nuclear magnetic resonance (NMR) spectra were recorded by an AVANCE 600 (Bruker, Germany). X-ray photoelectron spectroscopy (XPS) measurements were conducted using a ESCALAB 250Xi (Thermo Fisher Scientific Inc., USA). Scanning electron microscope (SEM) used in this research was a Gemini500 (ZEISS, Germany). X-Ray Diffraction (XRD) measurements were carried out using a D8-ADVANCE (Bruker, Germany). Ultraviolet–visible light (UV–vis) spectra were recorded by a UV-4150 (Hitachi, Japan).

### Statistical analysis

Data processing was conducted using Microsoft Excel (Microsoft 365). MestReNova was employed for the analyzation of NMR results. Origin 2024B was utilized for data plotting. GraphPad Prism 10.3 was used for statistical significance (P < 0.05) analysis.

## Results and Discussion

### Synthesis of CA@TAZn gel

**Scheme 1:**
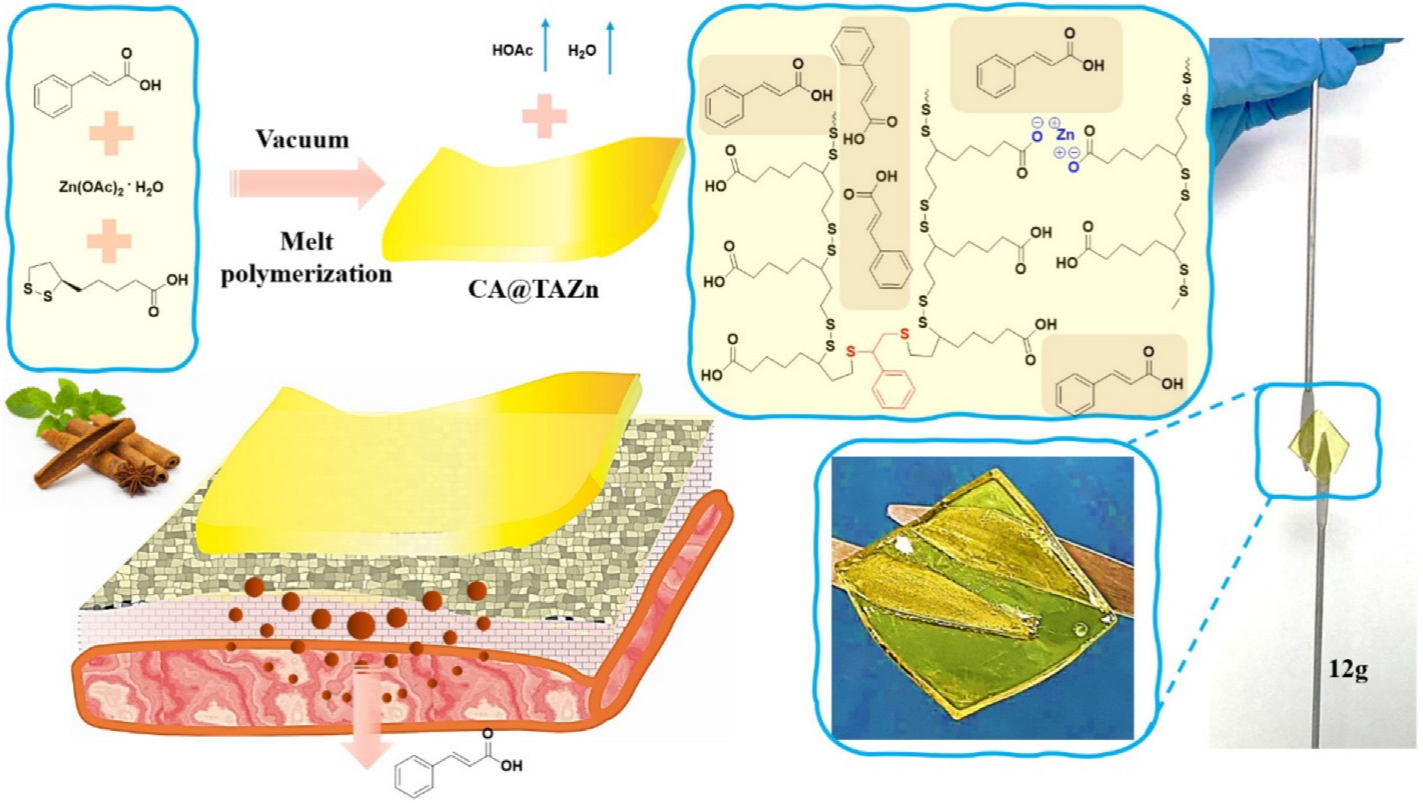
Schematic illustration of the preparation, the appearance, the chemical structure and the transdermal drug release property of CA@TAZn gel patch.

Straightforward loading of water insoluble or hydrophobic drugs into a vehicle by a non-solvent method is the main theme of this work. Generally, with an aim to combine various drug delivery system with CA, tactics such as host–guest interaction, polymer grafting, emulsification and nano encapsulation technology were adopted, ignoring the fact that the above tactics incurred tedious chemical routes, toxic organic solvent and hard quality control. Considering the wide solubility of low melting point organic sulfides and following the concept of thermal-grinding gel or thermal-grinding supramolecular network, CA was loaded into TA-zinc ion network to form a functional transdermal patch integrating multiple properties. Herein, the powder of CA, zinc acetate dihydrate and thioctic acid (TA) were mixed by grinding at first and then underwent thermal polymerization at 160 °C with a −0.1 MPa vacuum condition (Scheme 1). During the preparation procedure, the mixed powder was gradually melted with continuous agitated bubbles for further evenly mixing. The bubbles vanished while the mixture returned to normal atmospheric pressure. Finally, a homogeneous, transparent, flowable yellow solution was obtained in all CA@TAZn groups, which enabled to form a gel patch under cooling down to room temperature. The viscosity of TAZn and CA@TAZn ranged from 2.5 to 4.0 Pa•s at 160°C, respectively (Fig. S1). The liquid state of these melted mixtures with low viscosity facilitated debubbling and homogenization. This grinding-heating method is feasible in practical industrial scale-up production without any raw material modification, solvent consumption, precise stoichiometric feeding proportion or skilled operation means. The CA@TAZn gel patch turned out to be homogeneous and transparent, which showed adhesion toward stainless steel microspoons (12 g weight) owing to the multiple carboxyl-metal interactions. Without solvent phase, the construction of CA@TAZn followed a reinforced concrete structure in which both crosslinked network as well as CA aggregates spread throughout the gel. Therefore, it could be inferred that this gel could be potentially applied to release CA once adhered onto skin owing to its adhesion and its architecture. The storage stability of CA@TAZn gel patches with different feeding ratios was also studied during a 30-days storage (Fig. S2). In case of TAZn group, the gel stayed transparent with no sign of poly(TA) depolymerization. However, CA@TA group exhibited metastability since day 3, possibly caused by depolymerization of poly(TA) triggered by the radicals originated from photosensitive CA[17]. CA@TAZn-1, CA@TAZn-2 and CA@TAZn-3 with a constant CA/TA ratio of 1/6 showed desirable stability, revealing the introduction of zinc ions inhibited the metastability of poly(TA)-CA composites by restricting molecular activity. Furthermore, CA@TAZn-4 with an enhanced CA/TA ratio of 1/3 gradually became turbid since day 3. This phenomenon could be ascribed to the fact that the stabilizer effect of zinc ions was limited and that the excessive CA incurred higher chance for radical-induced poly(TA) metastability. Thus, TAZn, CA@TAZn-1, CA@TAZn-2 and CA@TAZn-3 groups were mainly studied. The pH values of all CA@TAZn gel patches, from 4.6 to 4.9 (Table 2), were in accordance with the pH value range of normal human skin (4.5–6.5).

**Table 2:** The pH values of CA@TAZn gel patches.

Sample name	pH value
CA@TAZn-1	4.66 ± 0.03
CA@TAZn-2	4.67 ± 0.03
CA@TAZn-3	4.87 ± 0.04

**Fig. 1:**
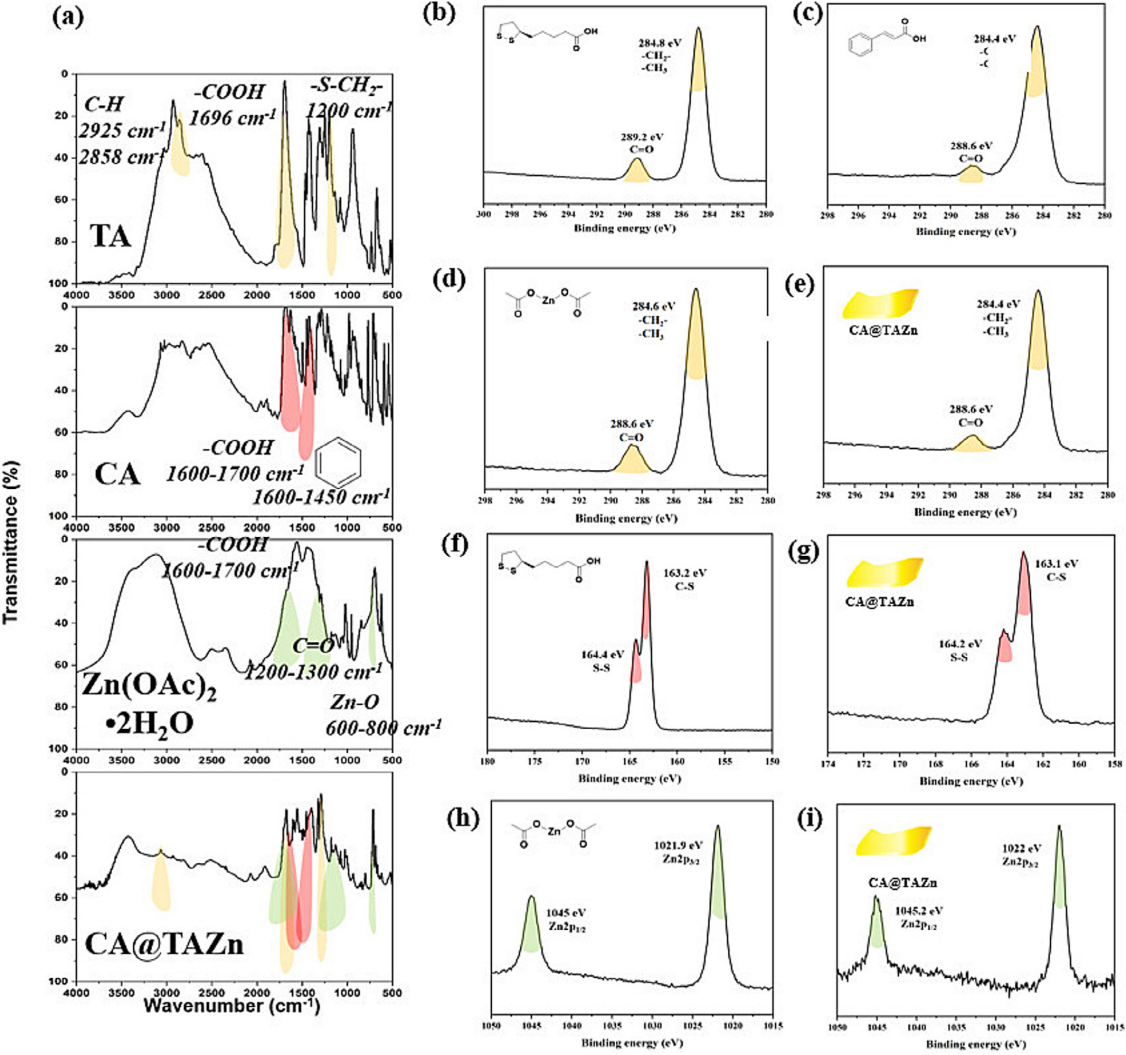
Chemical characterizations of CA@TAZn gel patch. (a) FT-IR spectra of TA, CA, Zn(OAc)_2_·2H_2_O and CA@TAZn; (b-e) XPS C1s spectra of TA, CA, Zn(OAc)_2_·2H_2_O and CA@TAZn; (f-g) XPS S2p spectra of TA and CA@TAZn; (h-i) XPS Zn2p spectra of Zn(OAc)_2_·2H_2_O and CA@TAZn.

The chemical structure of CA@TAZn was characterized by multiple characterization methods. From the prospect of FT-IR (Fig. 1a), CA@TAZn possessed aliphatic C–H peaks at 2932, 2859 cm^−1^ and S-CH_2_ peak at 1200 cm^−1^, derived from the main skeleton of poly(TA) after thermal-induced ring-opening polymerization (the yellow region in Fig. 1a). Moreover, carboxyl groups of TA at 1696 cm^−1^ and CA at 1650 cm^−1^ were also observed in CA@TAZn. Benzyl stretching at 1450–1600 cm^−1^ proved that CA was successfully introduced in CA@TAZn (the red region in Fig. 1a). Coupling peaks of carbonyl stretching/hydroxyl deformation vibration at 1200–1300 cm^−1^ and Zn-O peak at 600–800 cm^−1^ revealed that zinc ion existed in the final gel network (the green region in Fig. 1a). In view of the XPS C1s spectra, all ingredients as well as CA@TAZn had C=O peak at 288.6–289.2 eV and aliphatic C–H peak at 284.4–284.8 eV (Fig. 1b-e). S2p spectra demonstrated that both S-S peak at 164.4 eV and C-S peak at 163.1 eV were observed in TA and CA@TAZn. Z2p_1/2_ peak at 1045.0 eV and Z2p_3/2_ peak at 1022.0 eV revealed the incorporation of zinc ions in CA@TAZn. All the above results illustrated that TA, as a low melting point quasi solvent, was proper for loading CA and zinc ions simultaneously.

There is a fact that CA is a kind of natural compound that could convert to styrene when heated above 130 °C and a fact that thiyl radical produced by melted TA is high reactive towards olefins, whether CA reacts with TA under high temperature is the main focus of the gel chemical architecture. In view of the ^1^H NMR spectrum of thioctic acid-cinnamic acid copolymer (CA@TA), a new peak appeared at 2.82 pm (labeled 10 in Fig. S3) accounted for the methylene group formed by thiyl radical-alkene addition. Moreover, the reduction of integral area of carbon–carbon double bond (C=C) after reaction was also observed (labeled b and f in Fig. S3). The remaining ratio of CA could be calculated by the following equation:

$\text{Remaining ratio of CA = 1-}\frac{{\text{Sb}}_{\text{CA@TA}}\text{/}{\text{Sde}}_{\text{CA@TA}}}{{\text{Sb}}_{\text{CA}}\text{/}{\text{Sde}}_{\text{CA}}}$ or .$\text{Remaining ratio of CA = 1-}\frac{{\text{Sf}}_{\text{CA@TA}}\text{/}{\text{Sde}}_{\text{CA@TA}}}{{\text{Sf}}_{\text{CA}}\text{/}{\text{Sde}}_{\text{CA}}}$

where Sb_CA_ and Sf_CA_ were the C=C integral area originated from ^1^H NMR spectrum of CA, Sb_CA@TA_ and Sf_CA@TA_ were the C=C integral area originated from ^1^H NMR spectrum of CA@TA, Sde_CA_ and Sde_CA@TA_ were the benzyl hydrogen integral area originated from CA and CA@TA, respectively. Thus, the remaining ratio of CA was 88.3–90.9 % when the feeding ratio of CA/TA was 1/6. In other words, approximately 10 % CA underwent decarboxylation to form styrene, subsequently participating in thiyl radical-alkene addition with TA during the whole preparation procedure. ^13^C NMR studies on TA, CA and CA@TA were also carried out (Fig. S4-S6). New peaks at 26.4 ppm (labeled 10, 11 in Fig. S6) and 36.2 ppm (labeled 12, 13 in Fig. S6) showed up in CA@TA, which were corresponding to the enhanced electron cloud density caused by CA residues after thiyl radical-alkene addition. A peak at 50.3 ppm assignable to tertiary carbon (labeled 9 in Fig. S6) also appeared since the loss of conjugate effect between C=C and benzene ring after thiyl addition. Moreover, the main peaks of CA (labeled a-i in Fig. S5-S6) still existed in CA@TA, more intense than the newly emerging peaks, also proving that only a small amount of CA participated in the thiyl radical-alkene reaction. Based on the NMR results, this reaction process could be deduced as follows: Styrene derived from CA coexisted with thiyl radicals derived from TA at first. Then thiyl radical-alkene addition occurred to form a copolymer (Scheme S1). Overall, CA@TAZn was a dual-crosslinked network gel, simultaneously possessing CA-TA covalent crosslinking and TA-zinc ion physical crosslinking. Results from XRD indicated that all ingredients including CA, TA and Zn(OAc)_2_·2H_2_O gained intensive crystalline peaks (Fig. S7a-c). However, CA@TAZn gel patch followed an amorphous pattern without obvious peaks (Fig. S7d). Therefore, a homogenous uncrystalline polymer network was formed without polymer-drug phase separation, which further proved that the reinforced concrete structure contained poly(TA) and CA aggregates were stable under storage. SEM images demonstrated that the surface morphology of CA@TAZn gel patch was evenly flat in absence of caves for solvent absorption (Fig. S8ab). Moreover, energy-dispersive spectroscopy mapping exhibited that the elements including C, O, S and Zn were uniformly distributed throughout the gel. Thus, it turned out that the grinding mixing followed vacuum melting methods were beneficial for preparing a homogenous supramolecular network (Fig. S8c-g). Above all, the non-solvent method for obtaining CA@TAZn was feasible for drug loading and polymeric network crosslinking with desirable uniformity, reproducibility and operability.

### Basic properties of CA@TAZn gel

**Fig. 2:**
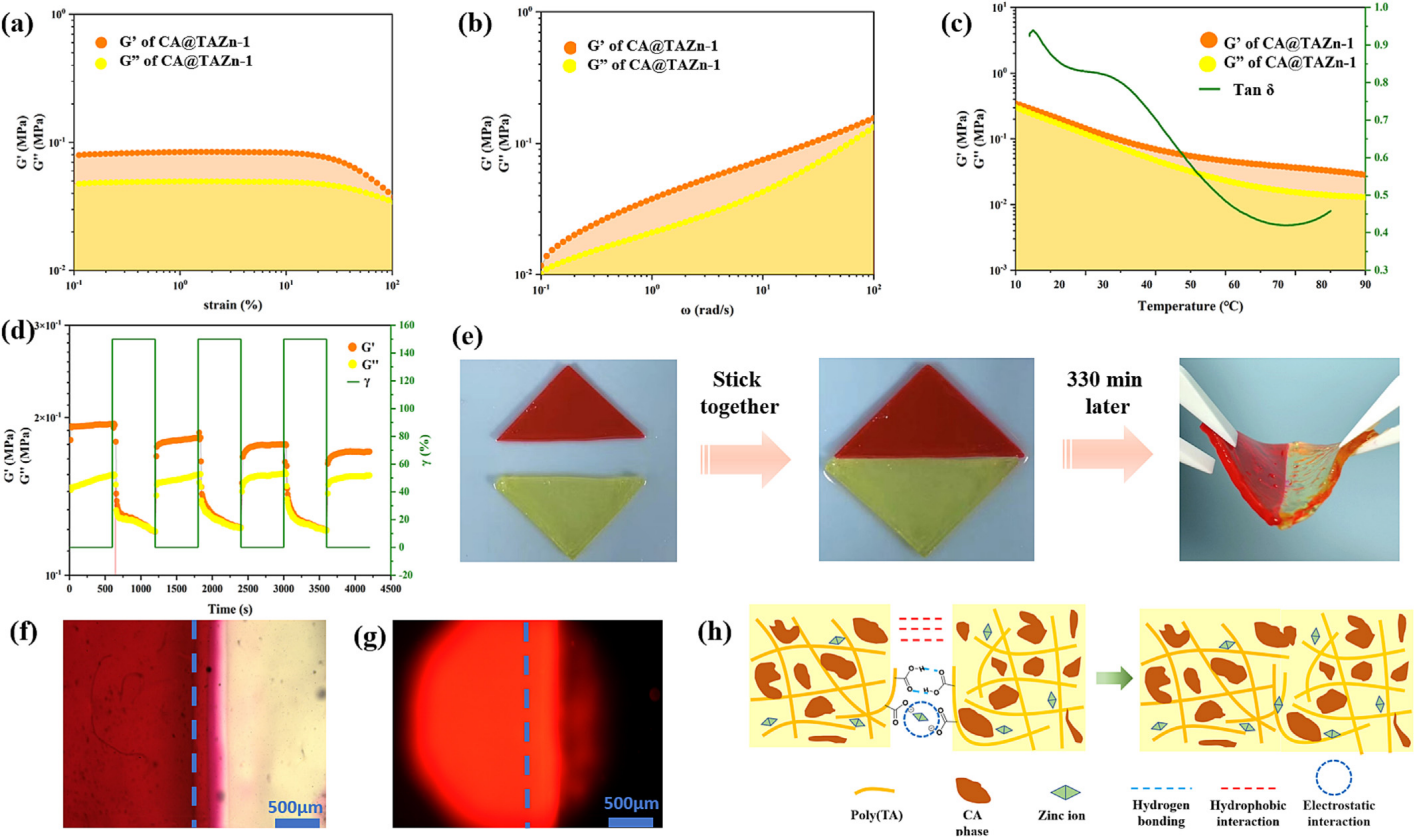
The rheological properties of CA@TAZn-1 gel patch. (a) Strain-sweep curve (25 ^°^C, ω = 10 rad/s); (b) Frequency-sweep curve (25 ^°^C, γ = 1 %); (c) Temperature-sweep curve (ω = 10 rad/s, γ = 1 %); (d) Repeated dynamic stepwise strain curve on the self-healing property of CA@TAZn-1 gel patch (γ = 1 % or 150 %,ω = 10 rad s^−1^); (e) Photos on the self-healing property of CA@TAZn-1 gel patch, half piece of the patch was stained by rhodamine B; (f) Photomicrograph on the self-healing border of CA@TAZn-1 gel patch; (g) Fluorescent photograph on the self-healing border of CA@TAZn-1 gel patch (scale bar = 500 μm); (h) Schematic illustration of the self-healing mechanism.

Though the CA@TAZn gel did not contain solvent phase, it had numerous gel-like properties such as viscoelasticity, self-healing and stretchability. As viewed from rheological strain-sweep, the storage modulus (G’) value of CA@TAZn-1 gel patch stayed constant at 0.08 MPa while the loss modulus (G’’) value of CA@TAZn-1 remained constant at 0.05 MPa when the strain ranged from 0.1 % to 30.0 % (Fig. 2a). G’ was higher than G’’ throughout the entire observation range, which manifested that the gel followed a semisolid performance with high gel network strength. Similarly, G’ also was higher than G’’ in the frequency-sweep (Fig. 2b). Interestingly, G’ value of CA@TAZn-1 gel patch increased with the rising frequency since the non-covalent interactions within the gel were frequency-dependent, which enhanced the gel network strength. Moreover, the rising frequency was also beneficial for the extension of flexible polymer chains within the gel, causing the increased G’’ value as well. The phenomenon of G’ > G’’ could also be observed in the temperature-sweep curve from 4 ^°^C to 90 ^°^C, which claimed that the gel network remained stable without melting (Fig. 2c). The tanδ value fell at a temperature range of 4-70^°^C because the cleavage of heat-labile H-bonds weakened the gel network strength. Considering the melting point of TA was 70 ^°^C, the dynamic disulfide exchange as well as the carboxyl group-zinc ion interaction could inhibit the gel from melting. Additionally, the CA@TAZn gel patch turned out to be recyclable since the gel cut slices could be melted at 160 ^°^C to form a uniform gel again as its original shape (Fig. S9). The rheological temperature-dependence of CA@TAZn enabled facile processing and molding, which is a typical feature of supramolecular bulk gels. A stepwise repeated dynamic strain assay was carried out to show that CA@TAZn-1 gel patch had excellent self-healing property, in which G’ > G’’ values at each odd step and G’ ≈ G’’ at each even step (Fig. 2d). Furthermore, macroscopic self-healing could be achieved since two pieces of CA@TAZn-1 gel patch, one normal piece and one rhodamine B-stained piece, reconstructed together after a 330 min storage (Fig. 2e). In view of microscope, both visible light micrograph and fluorescent micrograph proved that rhodamine B spread across the self-healing border and the border turned indistinct (Fig. 2fg). Physical crosslinking such as multi-carboxyl groups based hydrogen bonding as well as the carboxyl-zinc ion interactions accounted for the self-healing property of CA@TAZn gel (Fig. 2h). Compared to hydrogel contained continuous water phase, the non-solvent CA@TAZn gel has continuous drug storage phase so that the hydrophobic interactions were not neglectable. As CA was soluble in melted TA, hydrophobic interactions between aliphatic main skeleton of poly(TA) and CA aggregates also contributed to the self-healing process.

Another evidence for the hydrophobic nature of CA@TAZn was that the gel patches demonstrated water non-swelling property with a swelling ratio mostly lower than 10 % in water and PBS (Fig. S10-S12). Although ethanol was a good solvent for TA and CA, the gel patches could not absorb substantial amounts of ethanol with a swelling ratio lower than 10 %. This phenomenon could be explained by compact gel network in which poly(TA) was dual crosslinked by zinc ions as well as CA. Owing to the self-healing and anti-swelling property, CA@TAZn gel patch could be potentially used as a dermal patch that met versatile wearing environments such as showering, bleeding and alcohol wiping skin. Surface hydrophilicity was evidenced by the fact that the contact angles of TAZn, CA@TAZn-1, CA@TAZn-2 and CA@TAZn-3 gel patches varied from 65° to 75°, which could be ascribed to the inherent multi-carboxyl moieties from poly(TA) (Fig. S13). Once loaded with hardly water-soluble CA, the contact angles of CA@TAZn gel patches were slightly higher than that of TAZn.

The tensile properties of CA@TAZn gel patches were also conducted. From the viewpoint of tensile curves, all the samples including TAZn, CA@TAZn-1, CA@TAZn-2 and CA@TAZn-3 gained obvious mechanical yielding at the initial displacement (Fig. S14). Afterwards, the gel patches showed desirable ductility and finally reached cleavage. Four patterns can be summarized from tensile tests: (i) The tensile strength ascended with the increased zinc proportion and slightly lowered by CA loading. The mean tensile strength for TAZn, CA@TAZn-1, CA@TAZn-2 and CA@TAZn-3 was 0.52, 0.43, 0.81 and 0.95 MPa, respectively (Fig. S15); (ii) Contrarily, the maximum breaking elongation descended with the increased zinc proportion. CA@TAZn-1 reached the highest breaking elongation of 1976 ± 315 % (Fig. S16); (iii) Interestingly, the CA@TAZn-1 gel showed obvious self-adhesion in cyclic strain–stress test This tensile strength for ceasing self-adhesion was up to 0.17 MPa, much higher than that of yielding compared to other groups (Fig. S17a); (iv) The stretching of CA@TAZn gel patches would incur non-reversible deformation (Fig. S17d). For instance, CA@TAZn-1 gel patch emerged a 33 % creeping after a ten times cyclic strain–stress. In summary from the above four patterns, the initial yielding of gel patches resulted from the cleavage of carboxyl-zinc ion interaction. Afterwards, the mainchain of poly(TA) was free to be stretched and the gel tended rubber state. These destructions of non-covalent binding caused non-reversible deformation or gel fracture.

**Fig. 3:**
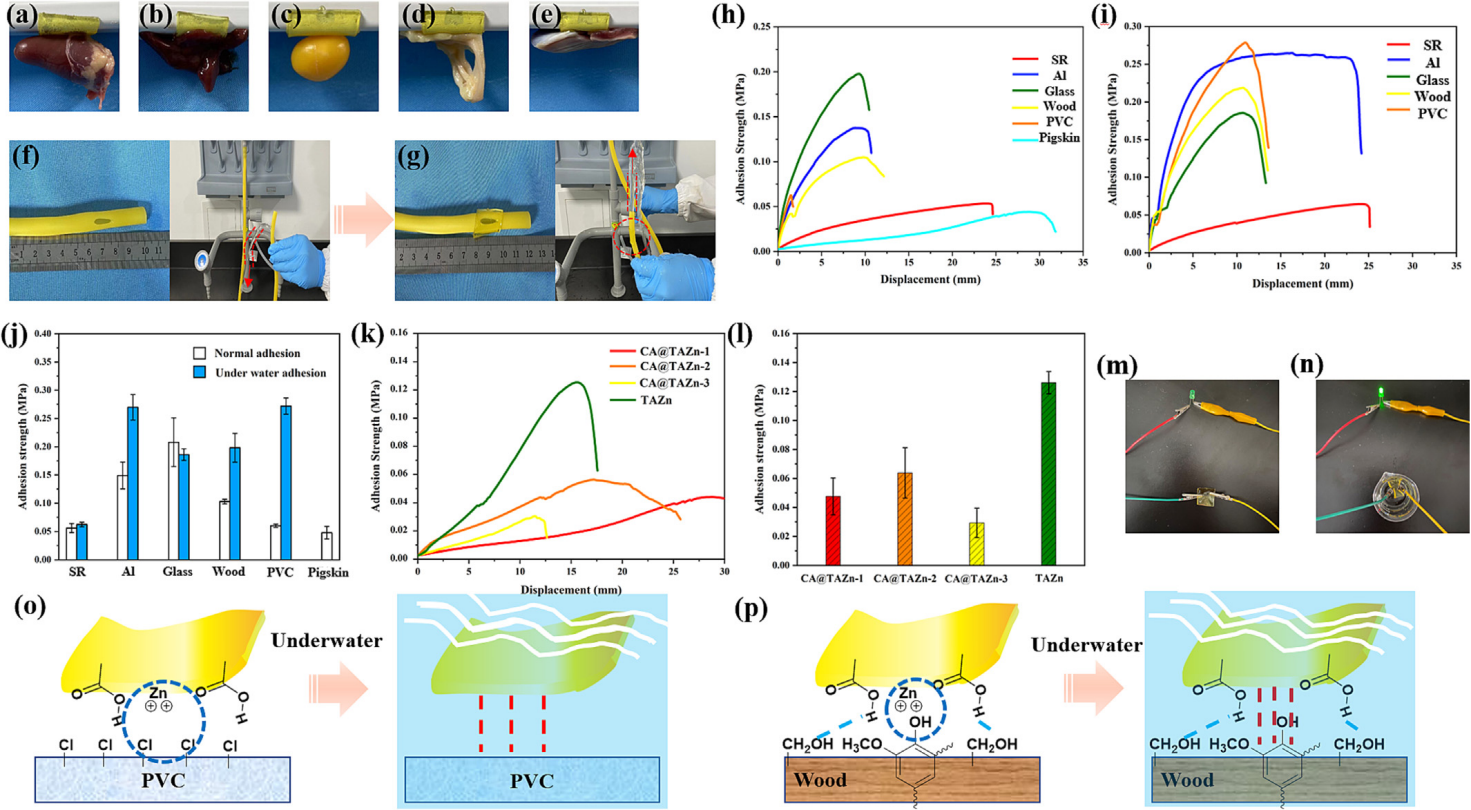
The adhesion property of CA@TAZn gel patches. (a-e) Chicken organs including heart, liver, yolk, intestines and gizzard hung from the CA@TAZn-1 gel patch, respectively; (f-g) CA@TAZn-1 gel patch was used as a waterproof tape for a rubber tube with a hole; (h) Representative lap-shear curves of the CA@TAZn-1 gel patch adhered silicon rubber, aluminum, glass, wood, poly(vinyl chloride) and pigskin after 24 h storage under atmosphere, respectively; (i) Representative lap-shear curves of the CA@TAZn-1 gel patch adhered silicon rubber, aluminum, glass, wood and poly(vinyl chloride) after 24 h storage underwater, respectively; (j) Normal and underwater adhesion strength of CA@TAZn-1 gel patch with various materials (n = 4); (k) Representative lap-shear curves of CA@TAZn gel patches adhered pigskin; (l) Adhesion strength of CA@TAZn gel patches with pigskin; (m) Non-conductive property of CA@TAZn-1 gel patch in atmosphere; (n) CA@TAZn-1 gel patch turned conductive underwater; (o) The adhesion mechanism between CA@TAZn and PVC; (p) The adhesion mechanism between CA@TAZn and wood.

Similar to other poly(TA)-based materials, CA@TAZn gel patch could also show affinity to a variety of materials owing to its multi-carboxyl based adhesion. The gel patch exhibited excellent adhesive properties towards chicken tissues such as heart, liver, yolk, intestines and gizzard (Fig. 3a-e). On one hand, the tissue contained abundant protein that allowed carboxyl-amide, carboxyl-amine and carboxyl-hydroxyl hydrogen bonding. On the other hand, tissue protein facilitated the hydrophobic interactions once contacted with the gel. Moreover, the application of CA@TAZn gel patch could expand to waterproof tape as it resisted water leakage from the hole of a rubber tube after adhesion (Fig. 3fg and Movie S1). Lap-shear tests for studying the CA@TAZn-based adhesion towards various materials were also carried out. After 24 h adhesion in atmosphere, CA@TAZn-1 gel patch showed the strongest adhesion towards glass slides with an adhesion strength approached 0.20 MPa (Fig. 3h). The gel was also proved to have desirable adhesion affinity to other materials since the adhesion strength for silicon rubber (SR) was 0.06 MPa, for aluminum (Al) 0.15 MPa, for wood 0.10 MPa, for poly(vinyl chloride) (PVC) 0.05 MPa and for pigskin 0.10 MPa. However, the underwater adhesion exhibited a different pattern from the normal state adhesion (Fig. 3ij). Both the adhesion strength of Al and PVC reached 0.27 MPa. Obvious enhancement of adhesion strength also occurred in wood underwater adhesion, which was up to 0.20 MPa. While small enhancement of adhesion strength showed up in SR underwater adhesion. On contrary, the underwater adhesion between CA@TAZn-1 and glass was weaker than normal state adhesion, reducing from 0.20 MPa to 0.18 MPa. Moreover, the CA@TAZn gel patch could also be utilized to adhere and lift a 200 g alloy weight underwater (Movie S2). The effect of feeding ratios on CA@TAZn-based pigskin adhesion was also studied via lap-shear tests, in which TAZn gel patch demonstrated the highest adhesion strength up to 0.12 MPa while CA@TAZn-3 patch showed the lowest adhesion strength up to 0.03 MPa (Fig. 3kl). Therefore, both CA and zinc ions lowered the pigskin adhesion because the inner gel supramolecular crosslinking weakened the multi-carboxyl-based adhesion. The reason why slightly higher pigskin adhesion strength of CA@TAZn-2 than that of CA@TAZn-1 was that the higher mechanical strength of CA@TAZn-2 gel allowed effective adhesion under longer tensile displacement. CA@TAZn-1 itself had no sign of electrical conductivity at a voltage of 9 V in normal state (Fig. 3m). Nonetheless, it seemed that the gel turned to be conductive underwater (Fig. 3n). Over all the phenomena on chemical structure, normal adhesion and conductivity, the variation in underwater adhesion strength could be explained as follows: (i) When the specimens were put underwater, the hydrophobic interactions caused ascending adhesion strength since underwater hydrophobic aggregation enhanced hydrophobic interactions in case of PVC (Fig. 3o); (ii) The escape of zinc ions from CA@TAZn gel patch entitled the conductivity underwater, which also increased multi-carboxyl hydrogen bonding interaction in case of Al; (iii) In an appropriate case of glass slides, nearly no hydrophobic interactions-based adhesion existed because of its hydrophilic surface. Moreover, the hydrogen bonding between CA@TAZn and glass slides was weakened, eventually led to reduced adhesion strength; (iv) Taking the chemical structure of lignin into account, multiple interactions such as zinc ion-phenolic electrostatic interactions, hydrogen bonding and hydrophobic interactions gave contribution to wood adhesion. The main reason for the enhanced adhesion strength underwater could be ascribed to the increased hydrophobic interaction (Fig. 3m). Viewing from these adhesion properties, the CA@TAZn not only acted as a wearable skin patch in clinical circumstances, but also showed potential usage in underwater adhesion or waterproof tape. The variation in adhesion strength during different time periods were also conducted (Fig. S18). The semi-solid gel property as well as the gradual formation of various van der Waals force at interfaces were accounted for the increased adhesion strength toward glass and pigskin at prolonged adhesion period.

### Biocompatibility, transdermal drug delivery and antibacterial property of CA@TAZn

**Fig. 4:**
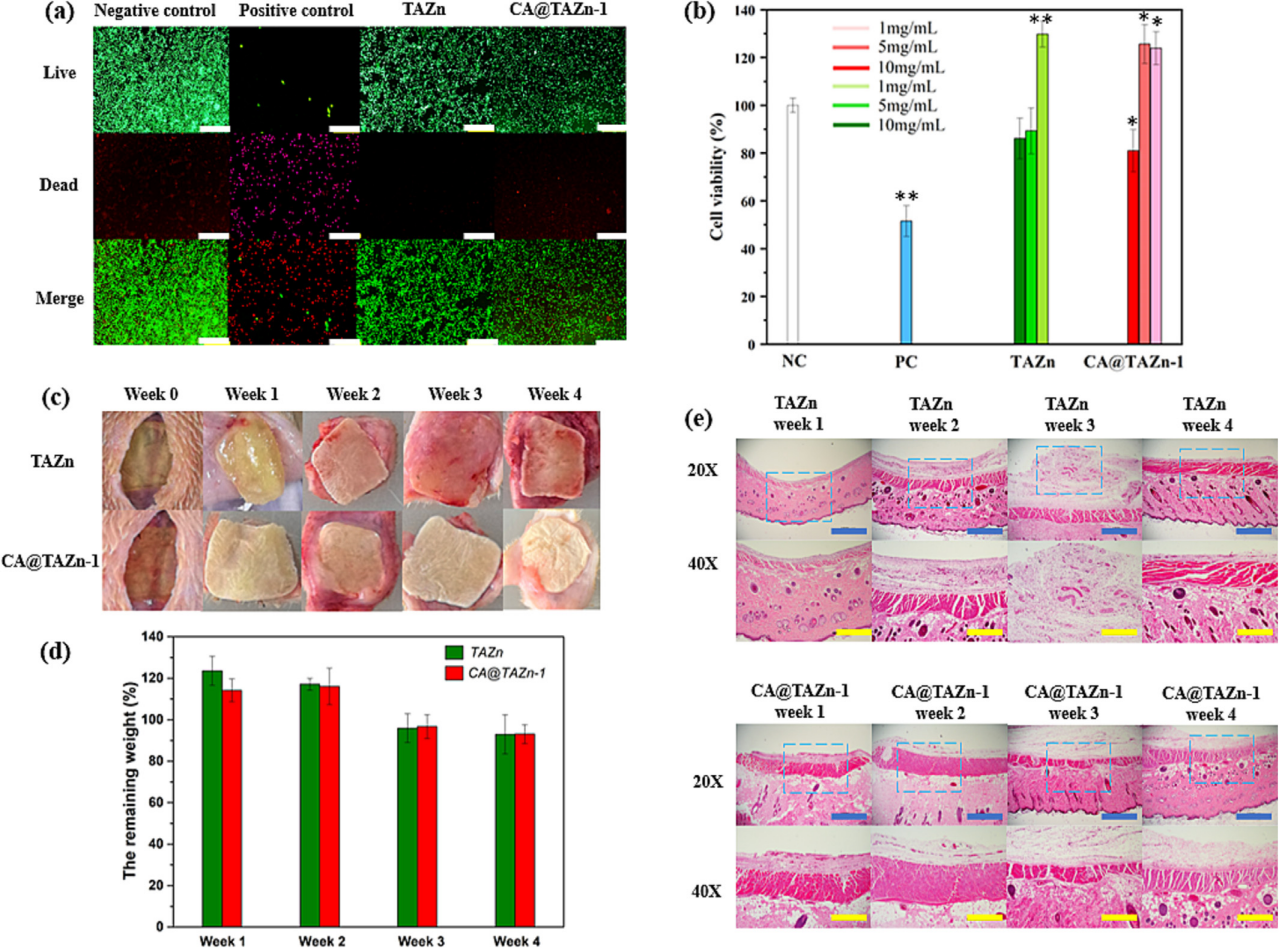
The biocompatibility of CA@TAZn gel patch. (a) Calcein AM/PI staining of NIH3T3 cells treated by negative control, positive control, TAZn and CA@TAZn after 24 h incubation; (b) Cell viability from CCK-8 assay (n = 3); (c) Photos on the gel patches (10 mm square) subcutaneously implanted into rats at different time points; (d) The *in vivo* degradation behavior of TAZn and CA@TAZn gel patches (n = 4); (e) H&E staining images for skin tissues surrounding gels at corresponding time points. (white scalebar = 200 μm, blue scalebar = 1000 μm, yellow scalebar = 500 μm, *P < 0.05, **P < 0.01). (For interpretation of the references to colour in this figure legend, the reader is referred to the web version of this article.)

The biosafety of CA@TAZn gel patch is a critical standard that affects its *in vivo* application as well as the corresponding curative effect. Though CA was proved to be antiproliferative towards most cancer cells, it preferred limited accumulation in normal cells[18]. Herein, the *in vitro* cytotoxicity of TAZn and CA@TAZn gel was investigated with NIH3T3 cells. Multitudinous cells were visualized by Calcein AM/PI staining LIVE/DEAD assays in all groups except the positive control group, in which TAZn and CA@TAZn groups showed neglectable difference (Fig. 4a). Cell viability viewed via CCK-8 test exhibited that all gel groups gained viability values above 80 % treated with a 10 mg/mL gel extract, further proving that the loading of CA showed minimal cytotoxicity towards NIH3T3 cells (Fig. 4b). Compared with the negative control, similar cell viability treated with diluted CA@TAZn gel extract, 1 or 5 mg/mL, could also be observed. The above results claimed that both CA and TAZn gel substrate owned desirable cell proliferation ability in case of NIH3T3 cells, which could be potentially used in drug delivery *in vivo* and wound healing. Furthermore, *in vivo* implantation of gel patches under physiological conditions was also carried out to study the corresponding biosafety and biodegradation. The transparent gels turned blur once implanted into tissue since the contacting of body fluid and the in-situ cell proliferation (Fig. 4c). The weight of all gels enhanced during the first two weeks and subsequently gradually reduced during week 3 and week 4, which was ascribed to the breakage of disulfide bonds within the gels stimulated by various thiol-containing compounds in physical condition (Fig. 4d). In TAZn group, H&E staining showed obvious accumulation of inflammatory cells at the sites directly approaching the gel surface during week 1 and week 2, manifesting inflammation aroused by transplantation rejection and anto-immune (Fig. 4e). Whereas the CA@TAZn group showed alleviated inflammation during the whole implantation process owing to the anti-inflammation performance of CA. Except the wearable performance, CA@TAZn gel could also be employed as an *in vivo* implanted polymer material for drug delivery with minimal transplantation rejection.

**Fig. 5:**
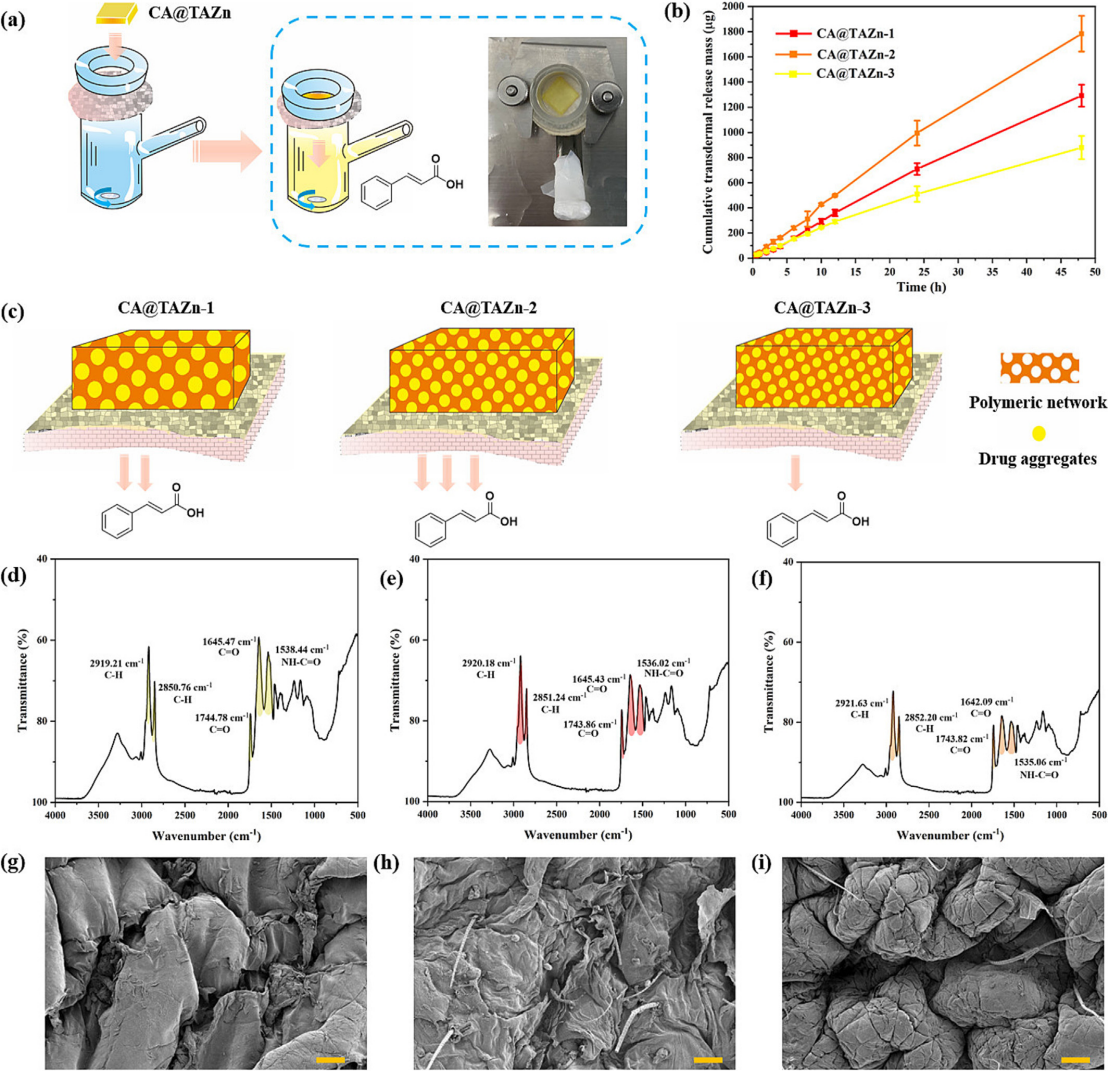
*In vitro* transdermal-HPLC measurements. (a) Schematic illustration of the transdermal CA release process; (b) Cumulative transdermal release mass of CA@TAZn gel patches across the porcine skin of Bama miniature pig (n = 3); (c) The CA release behavior effected by the density of polymer network; (d) ATR-FTIR spectrum of untreated rat skin; (e) ATR-FTIR spectrum of rat skin applied CA@TAZn gel for 24 h; (f) ATR-FTIR spectrum of rat skin applied CA@TAZn gel for 48 h; (g) SEM image of untreated rat skin; (h) SEM image of rat skin applied CA@TAZn gel for 24 h; (i) SEM image of rat skin applied CA@TAZn gel for 48 h (The scalebar was 50 µm).

It seemed that CA@TAZn without continuous solvent phase limited the CA release in practical uses. It was widely acknowledged that CA had a strong UV adsorption around 285 nm. The UV–vis spectra showed that CA@TAZn could gradually release CA when socked in PBS solution during 8 h observation (Fig. S19). Pointed at this issue, *in vitro* transdermal-HPLC assay was carried out. At first, the perfect linearity was detected from 1 to 500 µg/mL between concentration and peak area for CA, where the correlation coefficient was 0.9992 (Fig. S20). In this test, CA@TAZn-1, CA@TAZn-2 and CA@TAZn-3 gel patches were directly adhered onto the porcine skin of Bama miniature pig, incurring sustained CA release after a time lag up to 30 min (Fig. 5a). The CA release behavior of all CA@TAZn followed a first-order kinetic release since the linear curve slopes in the first 12 h were nearly the same as those of the whole 48 h (Fig. 5b). Therefore, the release rates of CA@TAZn-1, CA@TAZn-2 and CA@TAZn-3 gel patches were 27, 40 and 20 µg/h, respectively. Additionally, the permeation fluxes for the three formulations were 15.38 ± 0.99, 21.00 ± 1.58, and 10.17 ± 1.20 μg/cm^2^/h, respectively. Corresponding permeability coefficients were 0.72 ± 0.05, 0.98 ± 0.07, and 0.47 ± 0.06 (×10^-3^ cm/h), respectively (Table 3). As a result, CA@TAZn gels were proved to be drug container and the sustained CA release behavior of the gel patches was driven by concentration differences. The reason why CA@TAZn-2 achieved the highest release rate was explained by a polymeric network-drug aggregates mesh model (Fig. 5c). The network porosity of CA@TAZn-2 optimized the surface area of the drug phase, which resulted in higher release rate of CA@TAZn-2 than CA@TAZn-1. In case of CA@TAZn-3, densed network obstructed the overflow of drugs. Moreover, CA@TAZn-2 gel patch gained higher adhesion strength towards pigskin than other CA-loading patches, which revealed that CA@TAZn-2 gel patch had a maximum effective contact area when contacting flatness skin. Overall, the sustained release behavior of CA@TAZn gels enabled the application in external treatment for certain diseases.

**Table 3:** Permeability Parameters of CA@TAZn gel Formulations (Mean ± SD, n = 3).

Formulation code	J_SS_ (µg/cm^2^/h)	K_P_ (×10^-3^ cm/h)
CA@TAZn-1	15.38 ± 0.99	0.72 ± 0.05
CA@TAZn-2	21.00 ± 1.58	0.98 ± 0.07
CA@TAZn-3	10.17 ± 1.20	0.47 ± 0.06

Furthermore, ATR-FTIR analysis revealed that the characteristic absorptions of lipids occurred at 2919.21 cm^−1^ (CH_2_ asymmetric stretch), 2850.76 cm^−1^ (CH_2_ symmetric stretch), and 1744.18 cm^−1^ (C=O stretch). The characteristic absorption peaks of keratin appeared at 1645.47 cm^−1^ and 1538.44 cm^−1^ (amide I/II). The lipid characteristic peaks of C–H symmetric and C–H asymmetric vibrations exhibited a blue-shift, following CATAZn gel applying for 24 h and 48 h. Conversely, the characteristic peaks of the amide I and amide II bands of stratum corneum keratin showed a red-shift (Fig. 5def). Spectral shifts in these characteristic peaks reflected the molecular alterations in the stratum corneum. In detail, the blue shifting indicated a transition of lipid lateral accumulation from orthorhombic packing to an unstable hexagonal phase, progressing to a liquid-crystalline phases. The red shifting of the characteristic peak in the amide I band suggested keratin conformational transition from an alpha helical arrangement to a β-sheet structures, consequently destabilizing organization in the stratum corneum. The above information demonstrated that CA@TAZn gel enhanced transdermal drug delivery by lipid fluidization of lipids and by altering keratin conformation.

Observation through SEM revealed that the untreated rat stratum corneum, appeared relatively smooth and flat with a dense texture and slight wrinkles featuring dense, regularly arranged lipid layers with minimal inter-layer gaps. However, after applying of CA@TAZn gel for 24 h, the rat skin stratum corneum surface swelled, displaying increased thickness and distinct gaps between the lipid layers in the stratum corneum. Moreover, after 48 h application, morphological changes intensified in the morphology of the stratum corneum, with enlarged gaps between the thin lipid layers and lipid loosening. Epidermal lipid layers also detached and separated (Fig. 5ghi). These observations suggested that the use of CA@TAZn gel promoted drug penetration by altering the smoothness of the stratum corneum surface and gaps between lipid layers. Therefore, it was preliminarily concluded that the transdermal penetration mechanism of CA@TAZn gel was related to the structural changes of lipids and keratin in the stratum corneum, based on ATR-FTIR and SEM analysis.

**Fig. 6:**
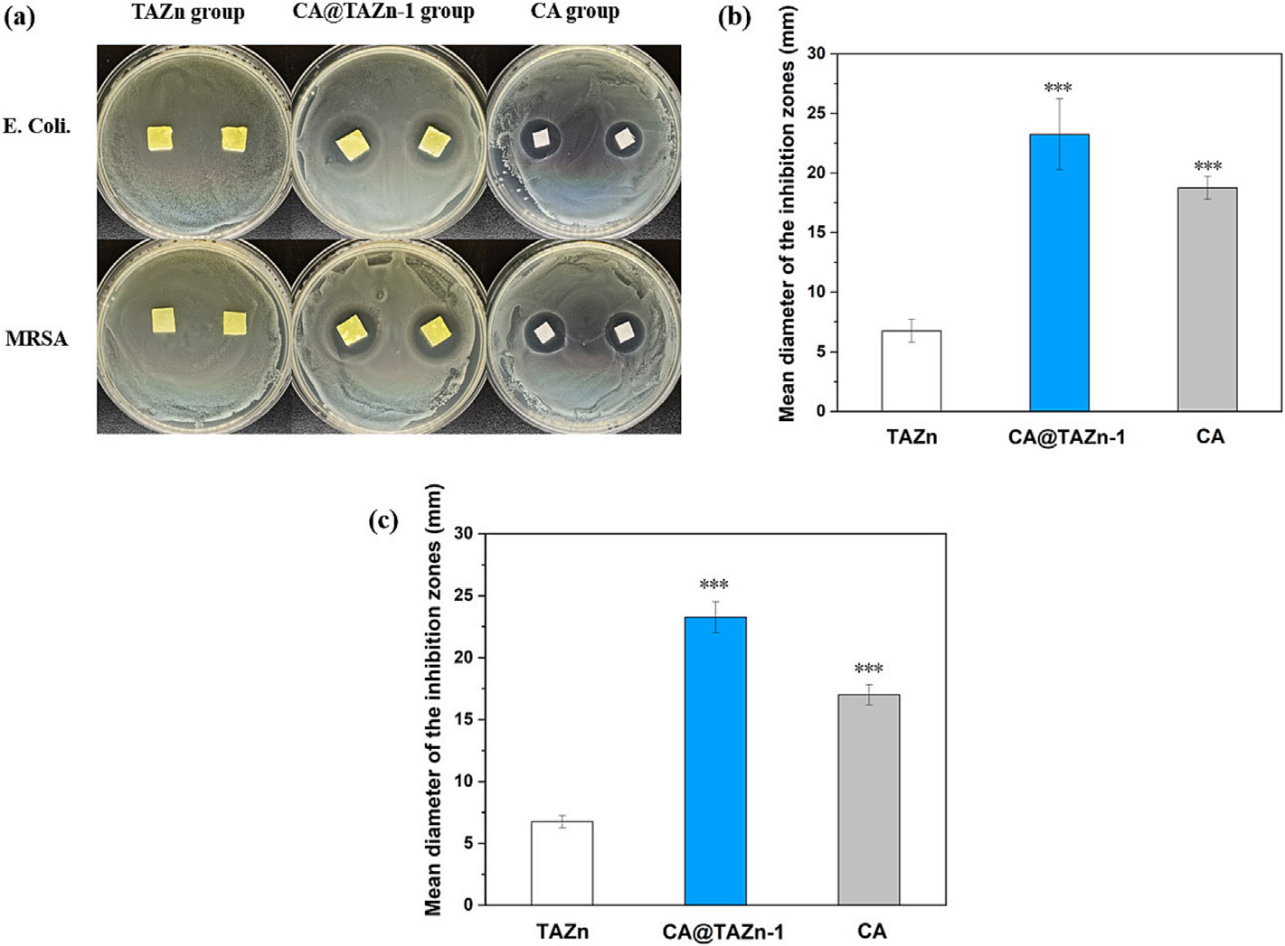
The antibacterial property of CA@TAZn gel patch. (a) Images on the inhibition zones for E. Coli. and MRSA; (b) The mean diameter of the inhibition zones for *E. Coli.*; (c) The mean diameter of the inhibition zones for *MRSA* (n = 4, ***P < 0.001).

Antibacterial property is quite a basic requirement for transdermal patches. In this point, TAZn and CA@TAZn-1 gel patches were subjected to antimicrobial inhibition zone tests, in which Gram-positive bacteria of *Methicillin resistant Staphylococcus aureus* (*MRSA*) and Gram-negative bacteria of *Escherichia coli* (*E. coli*) were used. As a result, the loading of CA substantially enhanced the antibacterial performance and the mean diameter of inhibition zones reached 23 mm (Fig. 6). Whereas the TAZn gel patch exhibited no antibacterial activity. The antimicrobial action of CA relies on the membrane disruption of bacteria.[32] The inhibition zone diameters of CA@TAZn-1 were slightly larger than those of CA. This phenomenon could be attributed to the prolonged pharmacodynamics through sustained-release of CA from the crosslinked gel network.

### The applications of CA@TAZn gel *in vivo*

In reference to the previous adhesion tests, CA@TAZn gel patches possessed excellent tissue adhesion and underwater adhesion that met the standard for trauma emergency patch. Thus, the hemostatic effect of the gel was also conducted via a SD rat liver hemostasis model, in which a part of the liver lobe was cut off to simulate acute trauma. The liver bleeding site without any treatment was used as blank control meanwhile gauze pressing was used as positive control. In contrast, TAZn and CA@TAZn gel patches were utilized as hemostatic material (Fig. S21ab and Movie S3). As a result, the hemostatic efficiencies of positive control, TAZn and CA@TAZn were 45 ± 12 %, 76 ± 8 % and 67 ± 11 %, respectively. Typically, both the TAZn and CA@TAZn gel arrested bleeding once covered the damaged liver. The interfacial binding affinity between gel patches and protein-rich tissue could be attributed to the multi-carboxyl based hydrogen bonding as well as the hydrophobic interactions (Fig. S21c). As CA has the effect of promoting blood circulation,[33] *trans*-tissue release of CA would be the main reason for the lowered hemostatic efficiency of CA@TAZn compared to that of TAZn. Despite CA in some degree inhibited hemostasis, the TAZn gel system still had potential in obtaining better bleeding ceasing property via further combining the adhesive polymeric network and hemostatic agents.

**Fig. 7:**
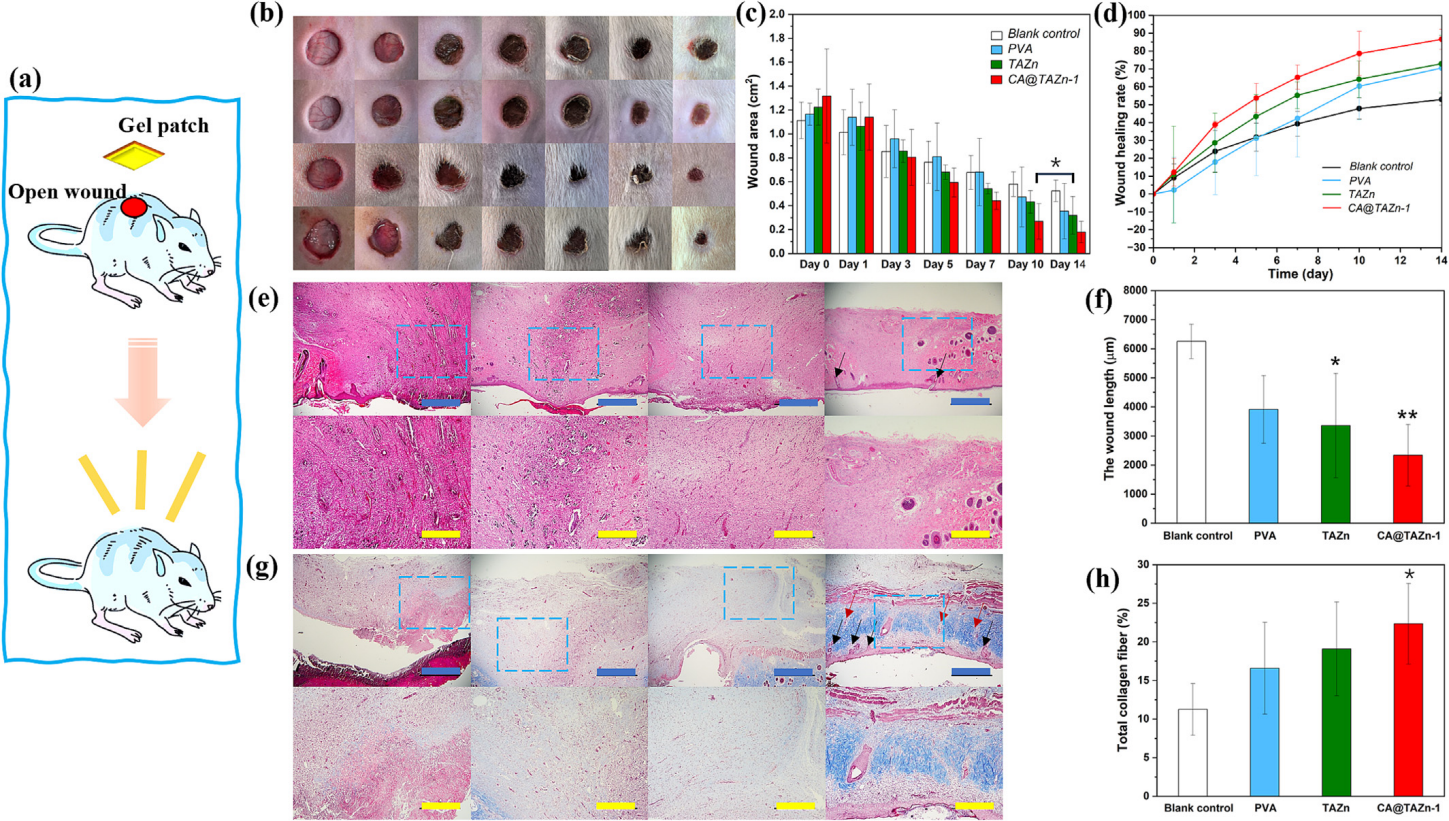
*In vivo* wound healing test. (a) Schematic illustration on the treatment of the whole layer skin defect via CA@TAZn gel; (b) Wounds after different treatments on 0th, 1st, 3rd, 5th, 7th, 10th and 14th day; (c) The variation of the wound areas in blank control, PVA hydrogel, TAZn gel and CA@TAZn-1 gel groups; (d) The wound healing rates of all groups; (e) H&E stained histological microimages showing the wound skin for treatments with different groups; (f) The wound length of all groups; (g) Masson-stained histological microimages showing collagen deposition for treatments with different groups; (h) Statistics of the collage-occupied area (blue scalebar = 1 mm, yellow scalebar = 500 μm, black arrows indicate the new hair follicles, red arrows indicate the newly formed blood vessels, *P < 0.05, **P < 0.01). (For interpretation of the references to colour in this figure legend, the reader is referred to the web version of this article.)

Combining the tissue adhesive of poly(TA)-based gel patch and clinical values of CA such as antibacterial, anti-inflammation and pain relief, CA@TAZn gel patch was utilized as a full- thickness wound healing therapy (Fig. 7a). Obvious wound contraction could be observed in CA@TAZn-1 group especially in day 7, 10 and 14 in contrast to blank control group (Fig. 7b). The initial wound area was 1.10–1.30 cm^2^, the average wound areas in CA@TAZn-1 group were 0.44 ± 0.07 cm^2^, 0.27 ± 0.14 cm^2^ and 0.18 ± 0.09 cm^2^ in day 7, day 10 and day 14, respectively (Fig. 7c). Without CA loading, PVA hydrogel and TAZn gel showed similar curative effect, less effective than CA@TAZn-1 gel. While the average wound healing rates were 53 ± 3 %, 71 ± 17 %, 73 ± 16 % and 87 ± 6 % for blank control, PVA, TAZn and CA@TAZn-1 groups, respectively (Fig. 7d). The above results proved that the adhesive poly(TA) substrate could promote wound healing since its desirable biocompatibility as well as its bioadhesive property for wound sealing. Moreover, further enhancement of therapeutic effect concerning CA@TAZn gel patch could be ascribed to the transdermal release of CA that inhibited bacterial infection meanwhile promoted tissue regeneration.

In viewpoint of haematoxylin and eosin (H&E) stained wound tissues, the blank control group manifested obvious inflammatory infiltration with incomplete wound bed in day 14 (Fig. 7e). By contrast, the wound tissue obtained from CA@TAZn-1 group approximated to normal epidermal tissue with less inflammatory cells, newly formed hair follicles, condensed granulation tissues and favourable vascularization, which revealed that CA@TAZn gel was beneficial for wound healing due to the anti-inflammation effect of CA. Meanwhile, the wound length CA@TAZn-1 group, up to an average value of 2.3 mm, was the smallest among other groups (Fig. 7f). Furthermore, Masson trichrome staining was employed to investigate collagen fibre remodelling in the process of wound healing (Fig. 7g). The blank control, PVA and TAZn group exhibited limited collagen fibre. However, CA@TAZn-1 group showed abundant collagen fibre deposition and several mature hair follicles on the 14th day. The total collagen fibre content in the wound bed treated with CA@TAZn-1 gel reached 22.3 ± 5.2 % (Fig. 7h). All the above-mentioned results revealed that CA@TAZn gel was capable of dealing with open wounds or tissue traumas owing to the following advantages: (i) The preparation of CA@TAZn gel allowed adjustable size and shape, which was less time-consuming and facile to scale-up; (ii) This gel is biocompatible with skin tissue and the wound healed more completely than those without drug loading; (iii) The intrinsic lipophilic polydisulfide network was promising as a platform for multiple water-insoluble drug loading aimed at various clinical conditions; (iv) The antiswelling property and the underwater adhesive property of CA@TAZn gel took priority over other therapies in wound healing since the ever-changing wearing environment.

**Fig. 8:**
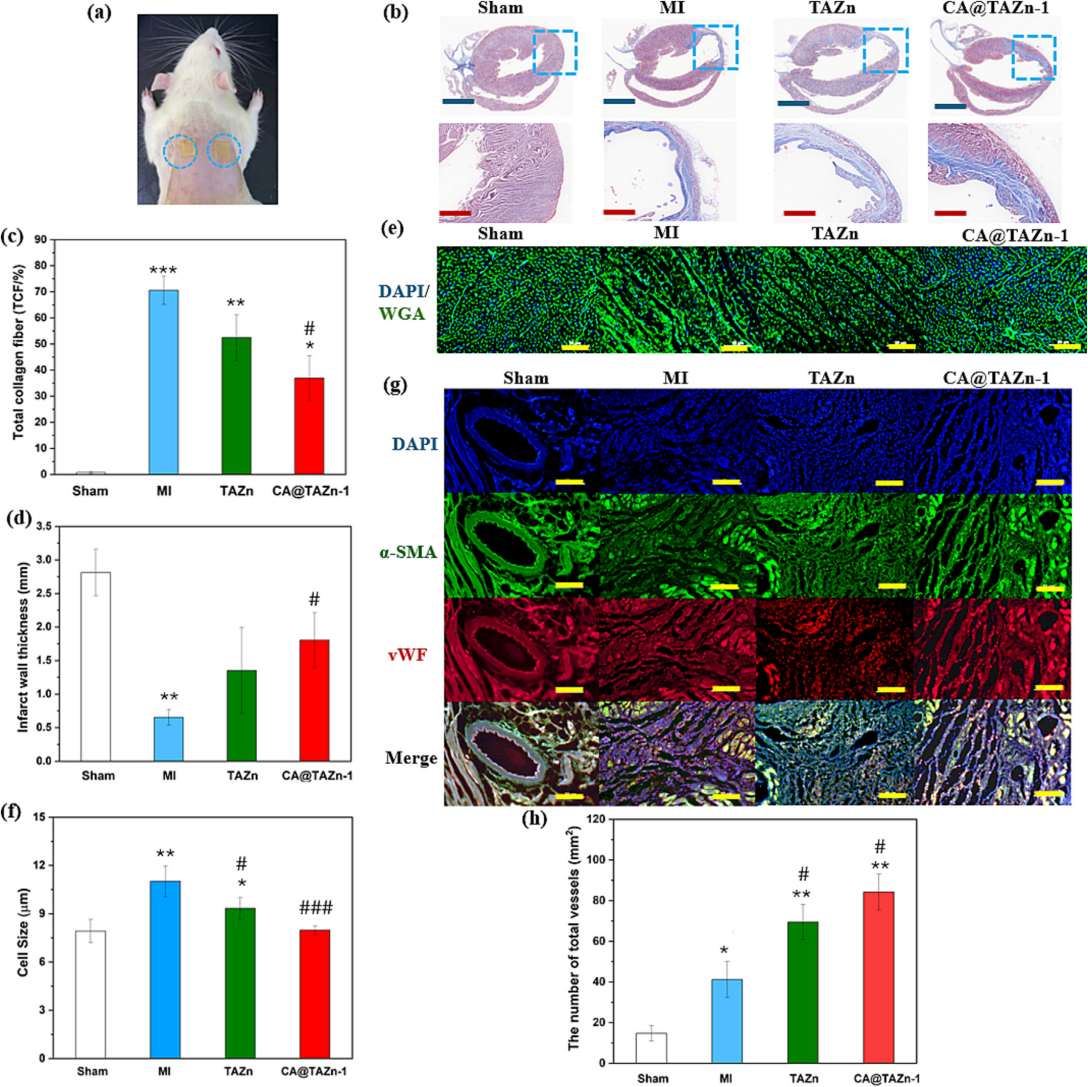
The therapeutic effect of CA@TAZn gel in myocardial infarction model. (a) Photogram on the acupoint application of CA@TAZn-1 gels; (b) The fibrous tissue (blue) and myocardium (red) of sections of hearts indicated by Masson trichrome staining (n = 4); (c) The proportion of total collagen fibers from each group deduced from Masson trichrome staining (n = 4); (d) Statistical analysis of infarct wall thickness of the infarcted heart from each group depended on Masson trichrome staining (n = 4); (e) Wheat-germ agglutinin (WGA) staining exhibited myocyte size; (f) Statistical analysis of the myocyte size based on WGA staining (n = 4); (f) vWF/α-SMA immunostaining staining displayed the infarct area, microvessels and arterioles from each group; (h) The number of total vessels (blue scalebar = 6 mm, red scalebar = 1 mm, yellow scalebar = 50 μm, *P < 0.05, **P < 0.01, ***P < 0.001 compared to sham-operated group, ^#^P < 0.05, ^##^P < 0.01, ^###^P < 0.001 compared to myocardial ischemia group). (For interpretation of the references to colour in this figure legend, the reader is referred to the web version of this article.)

CA and its derivatives have been proved to be effective in acute myocardial ischemia, which follows a mechanism based on descending serum levels of CK-MB, LDH, TNF-α and IL-6, and increasing serum NO activity.[34] Bearing transdermal drug delivery and anti-inflammation properties, CA@TAZn gel was promising in treatment of myocardial ischemia (MI). Herein, a SD rats MI model through left anterior descending artery was established and CA@TAZn gel acted as a transdermal patch adhered onto BL15 acupuncture points for a 6 days therapy (Fig. 8a). The cardiac fibrous tissue content from sham group, MI group, TAZn group and CA@TAZn-1 group were observed using Masson trichrome staining (Fig. 8b). High proportion of collagen fiber in MI group manifested the most severity of cardiac function decline. The proportions of total collagen fiber were 71 ± 5 %, 52 ± 9 % and 37 ± 9 % for MI group, TAZn group and CA@TAZn-1, respectively. Meanwhile, the infarct wall thicknesses were 2.8 ± 0.3 mm, 0.6 ± 0.1 mm, 1.4 ± 0.6 mm and 1.8 ± 0.4 mm for sham group, MI group, TAZn group and CA@TAZn-1, respectively. In comparison with MI group and TAZn group, decreased fibrosis could be observed in CA@TAZn group. Furthermore, quantified analysis revealed the decreased collagen fiber as well as the increased left ventricular thickness in CA@TAZn treated hearts in contrast to MI group and TAZn group (Fig. 8cd). Inhibition on hypertrophy of cardiomyocytes indicated the therapeutic effect of CA@TAZn gel patch, which is in relevance to restoration of cardiac function. Thus, wheat-germ agglutinin (WGA) staining was involved to observe the degree of cardiac hypertrophy in each group (Fig. 8e). As a result, sham group, MI group, TAZn group and CA@TAZn-1 group gained a cell size value up to 7.9 ± 0.7 μm, 11.0 ± 1.0 μm, 9.3 ± 0.7 μm and 8.0 ± 0.3 μm, respectively. After 6 days post-MI, the cell size of cardiomyocytes obviously ascended in the MI and TAZn groups, whereas the cell size of cardiomyocytes in CA@TAZn group was analogous to that in sham group (Fig. 8f). Moreover, generally distinct continuous cross-sectional patterns could also be observed in sham group and CA@TAZn group, indicating the resistance of cardiomyocytes hypertrophy. The supply status of blood in the infarct area is related to the survival of residual myocardial cells and further cardiac function restoration. With an aim to inspect the angiogenesis within the infarct area, vWF staining was used to visualize the microvessels, while vWF/α-SMA staining was used to visualize the arterioles (Fig. 8g). The numbers of total vessels per square millimetre were 41 ± 9, 70 ± 9 and 84 ± 9 for MI group, TAZn group and CA@TAZn-1 group, respectively. Sparce arteriole structure existed in MI and TAZn group. In contrast, dense and continuous microvessels and arterioles could be observed in CA@TAZn group. Statistical analysis on the total number of blood vessels demonstrated that CA@TAZn group, with the highest number of blood vessels up to 90 mm^2^, could substantially induce vasculation for cardiac function restoration (Fig. 8h). From the above results, CA@TAZn gel was proved to be effective in the treatment of acute myocardial ischemia owing to the following aspects: (i) CA@TAZn gel was capable of resisting ventricular remodeling and protecting heart from rupture during therapy process; (ii) The transdermal delivery of CA from CA@TAZn gel alleviated the oxidative damage and inflammatory damage of cardiomyocytes in the lesion location, promoting the procedure of angiogenesis; (iii) From the therapeutic effect of TAZn group, zinc ions also positively affected angiogenesis and cardiac tissue regeneration though this impact was limited; (iv) Last but not least, as a transdermal patching, CA@TAZn gel could conveniently administer medication, which skipped the potential discomfort from *in vivo* implantation or injection.

The comparison between CA@TAZn gel system and other CA loading systems was summarized in Table S1, the superiority of this transdermal drug delivery system lay in high CA loading capacity, low-cost raw material and easy solvent −free preparation method.

## Conclusion

Though hydrogel has shown great application prospects in biomedical, flexible electronics, soft robotics and other related fields, limitations such as dehydration aging and hard solublization for water-insoluble drugs should not be ignored. Efforts should be made for exploring new dosage forms and this study laid emphasis on non-solvent gel system. In this work, building blocks such as TA, CA and zinc acetate dihydrate resulted in a new non-solvent gel for CA loading. The concerning synthetic route was facile, robust and low-cost. The feature of the gel architecture not only relied on the polymeric network-CA aggregates based continuous phase, but also relied on multiple supramolecular interactions caused by zinc ions, abundant carboxyl groups and hydrophobic moieties. This gel had a tensile strength of 0.43–0.95 MPa and a maximum breaking elongation of 1000–2000 %. While the gel also exhibited anti-swelling and recyclability properties. The gel followed a homogeneous pattern viewed by SEM and XRD characterization. NMR results proved the dual-crosslinked network of the gel. The semisolid nature as well as the self-healing properties of the gel was revealed by rheological tests. Lap-shear tests illustrated that the gel possessed wide-scope adhesion towards different materials, in which the gel tended a better affinity to Al and PVC underwater with the adhesion strength over 0.25 MPa. The gel was proved to be antibacterial and biocompatible in perspective of E. coli/MRSA antibacterial zone experiment and NIH3T3 cells culture. From the viewpoint of *in vitro* transdermal-HPLC method, the gel enabled sustained delivery of CA with a release rate of 20–40 μg/h. According to the pharmaceutical activity of CA, the gel could also be applied to wound healing, hemostasis and myocardial ischemia. In prospect, in accordance with the idea that gel facilities water-insoluble drug loading meanwhile improved MI therapeutic effect, the non-solvent gel provided a new solution. In further research works, targeting complex and diverse diseases, we will focus on other thermal-polymerization gel systems for the loading of hydrophobic drugs or water-insoluble drugs.

## Compliance with ethics requirements

This research contained no human experiment. All animal studies were conducted in reference to the National Institutes of Health Laboratory Animal Care and Use Guidelines (NIH Publication No. 85-23 Rev. 1985) and experiments were approved by the Animal Ethics Committee of Shandong University of Traditional Chinese Medicine (Grant No. SDUTCM20230222302).

## CRediT authorship contribution statement

**Xi-xi Xiang:** Formal analysis, Data curation, Writing – original draft. **Qing-chang Xia:** Writing – original draft. **Xiao-bin Zhang:** Methodology. **Yu-wei Shi:** Formal analysis. **Ying-ying Yu:** Formal analysis. **Pei-jie Wang:** Formal analysis. **Feng-jun Ma:** Software. **Min Shen:** Validation. **Lin-lin Zhang:** Investigation. **Chen Chen:** Writing – review & editing, Supervision, Project administration, Funding acquisition. **Meng-zhen Xing:** Visualization, Project administration. **Qing-hua Cui:** Supervision. **Yu-ning Ma:** Resources. **Ting-ting Zheng:** Writing – review & editing, Project administration. **Xiao Yang:** Conceptualization.

## Declaration of competing interest

The authors declare that they have no known competing financial interests or personal relationships that could have appeared to influence the work reported in this paper.
