# Supplementary material for: Non-solvent cinnamic acid-based gel patch for transdermal drug delivery
Source: J Adv Res. 2025 Aug 8;83:695–711. doi: 10.1016/j.jare.2025.08.008 (PMC13154645; doi:10.1016/j.jare.2025.08.008)
Supplement: Supplementary Data 1 [file mmc1.docx]

**Supporting Information to “Non-Solvent Cinnamic Acid@Thioctic Acid-Zinc Ion Gel for Multiple Clinical Applications”**

**Xi-xi Xiang†^1^;** **Qing-chang Xia†^1^;** **Xiao-bin Zhang^1^; Yu-wei Shi^1^; Ying-ying Yu^1^; Pei-jie Wang^1^;** **Feng-jun Ma^1^;** **Min Shen^1^;** **Lin-lin Zhang^1^;** **Chen Chen*^1^;** **Meng-zhen Xing*^1^;** **Qing-hua Cui*^1^;** **Yu-ning Ma*^1^;** **Ting-ting Zheng*^1^;** **Xiao Yang*^2,3^**

^1^Shandong University of Traditional Chinese Medicine, Jinan 250355, China.

^2^The First Affiliated Hospital of Shandong First Medical University (Shandong Qianfoshan Hospital), Jinan 250014, China.

^3^Tianjin Medical University, Tianjin 300203, China.

†These authors contributed equally to this work.

*Correspondence to: C. Chen, M. Z. Xing, Q. H. Cui, Y. N. Ma, T. T. Zheng and X. Yang (E-mail: 21129008@zju.edu.cn; mengzhen@mail.ipc.ac.cn; cuiqinghua1122@163.com; myning0405@163.com; ttz10_10@163.com and 2485@sdhospital.com.cn).


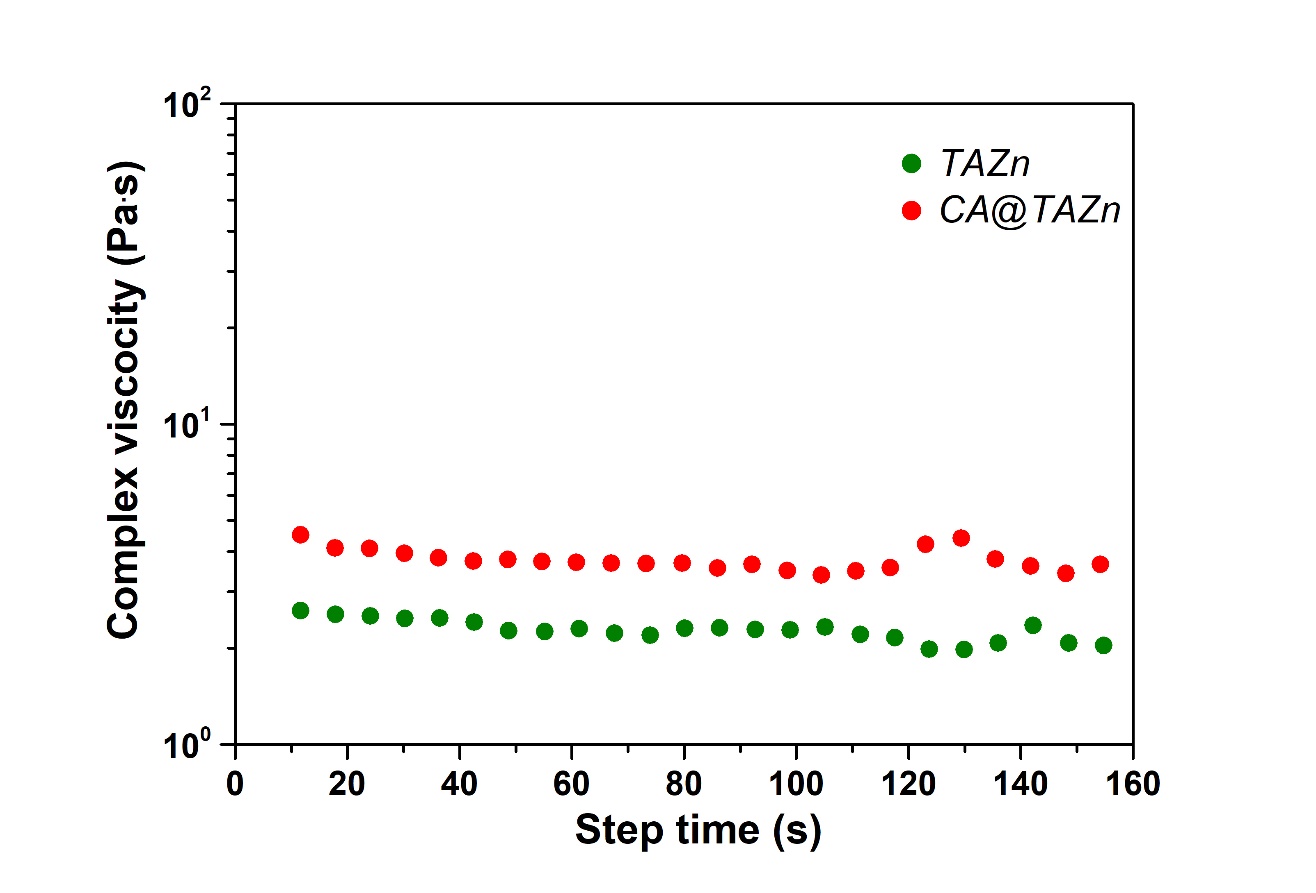


**Figure S1** Complex viscosity of melted TAZn and CA@TAZn-1 recorded by rheology at 160 °C (γ = 0.1%, ω = 10 rad s^−1^).


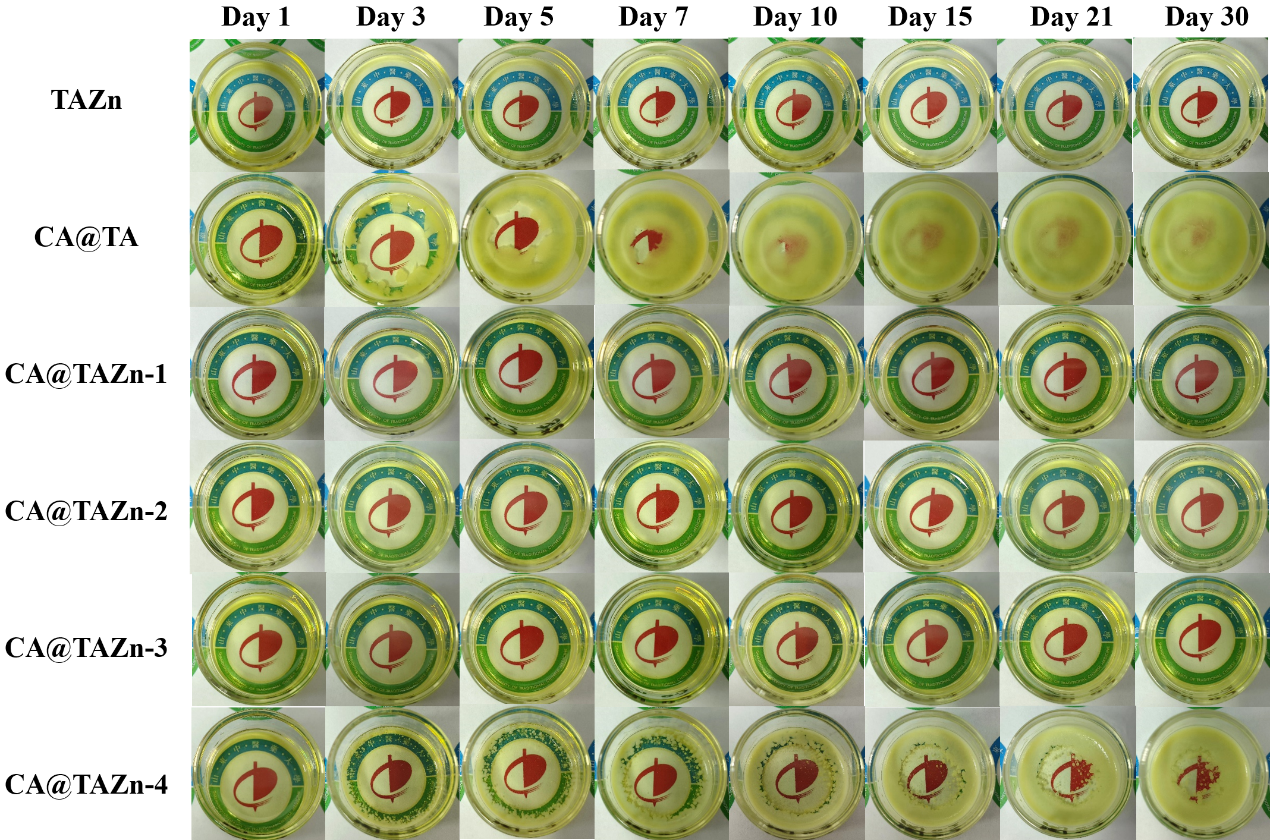


**Figure S2** Photos of CA@TAZn patches in quartz culture dishes during a 30-days storage.


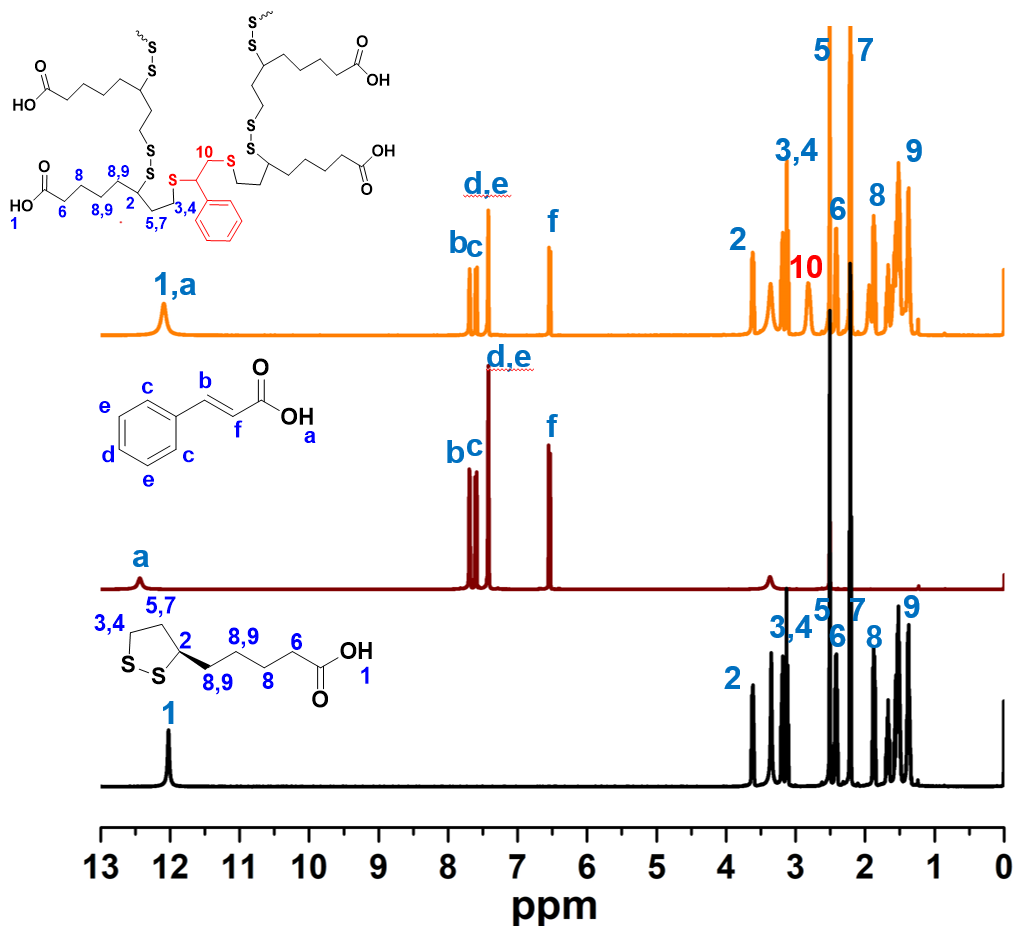


**Figure S3** ^1^H NMR spectra of thioctic acid, cinnamic acid and thioctic acid-cinnamic acid copolymer (CA@TA), the solvent was d_6_-DMSO.


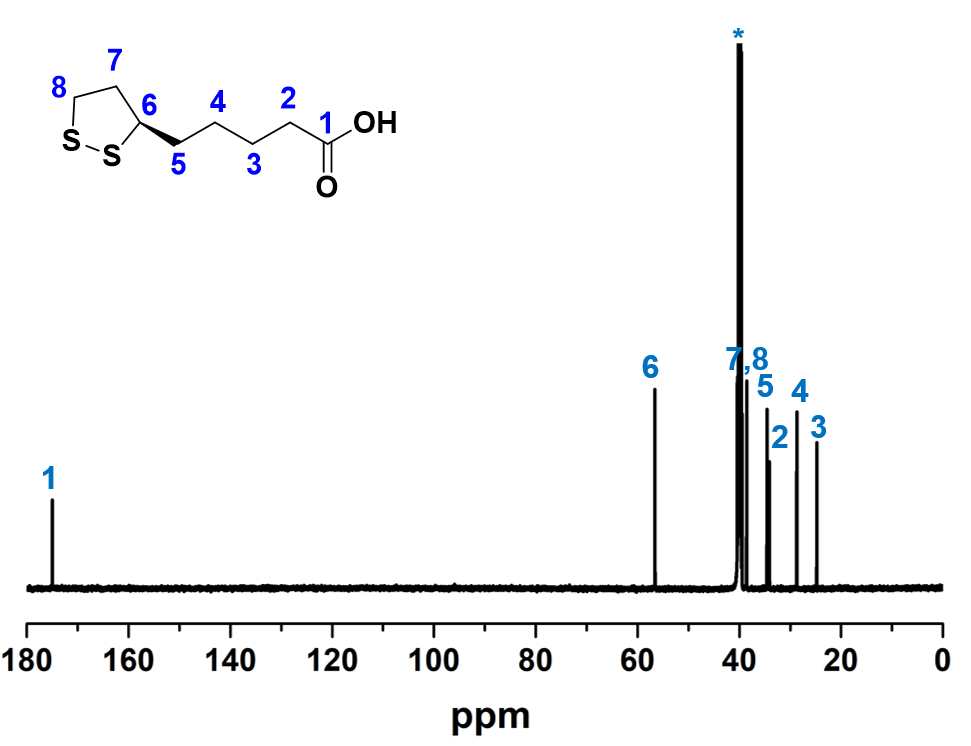


**Figure S4** ^13^C NMR spectrum of thioctic acid, the solvent was d_6_-DMSO.


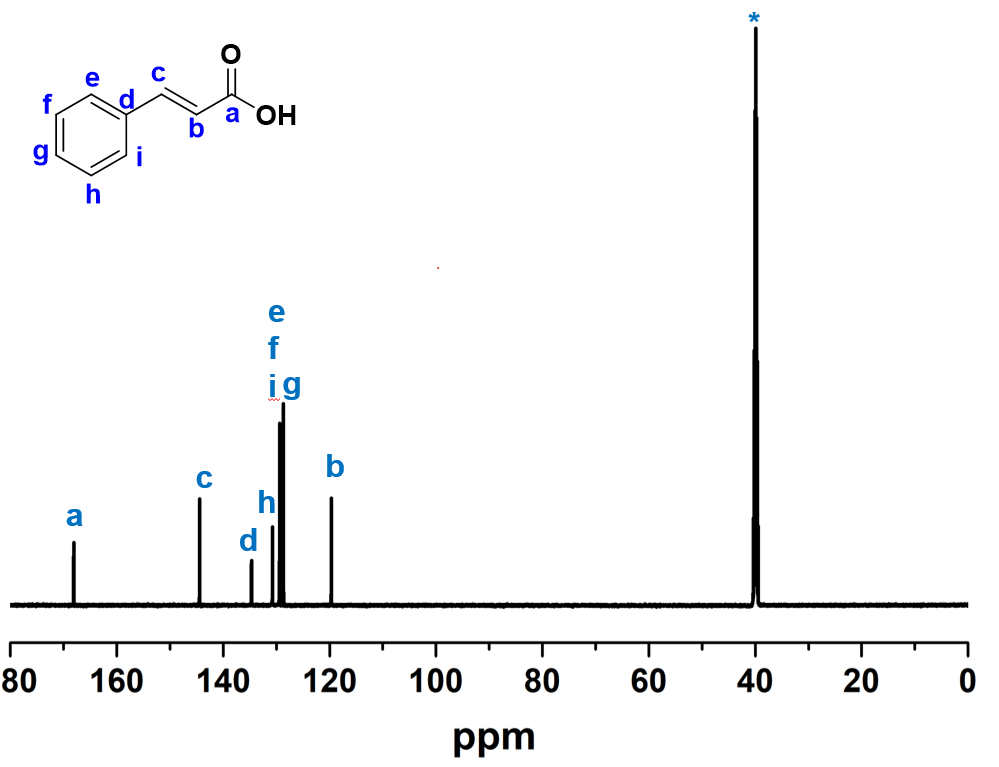


**Figure S5** ^13^C NMR spectrum of cinnamic acid, the solvent was d_6_-DMSO.


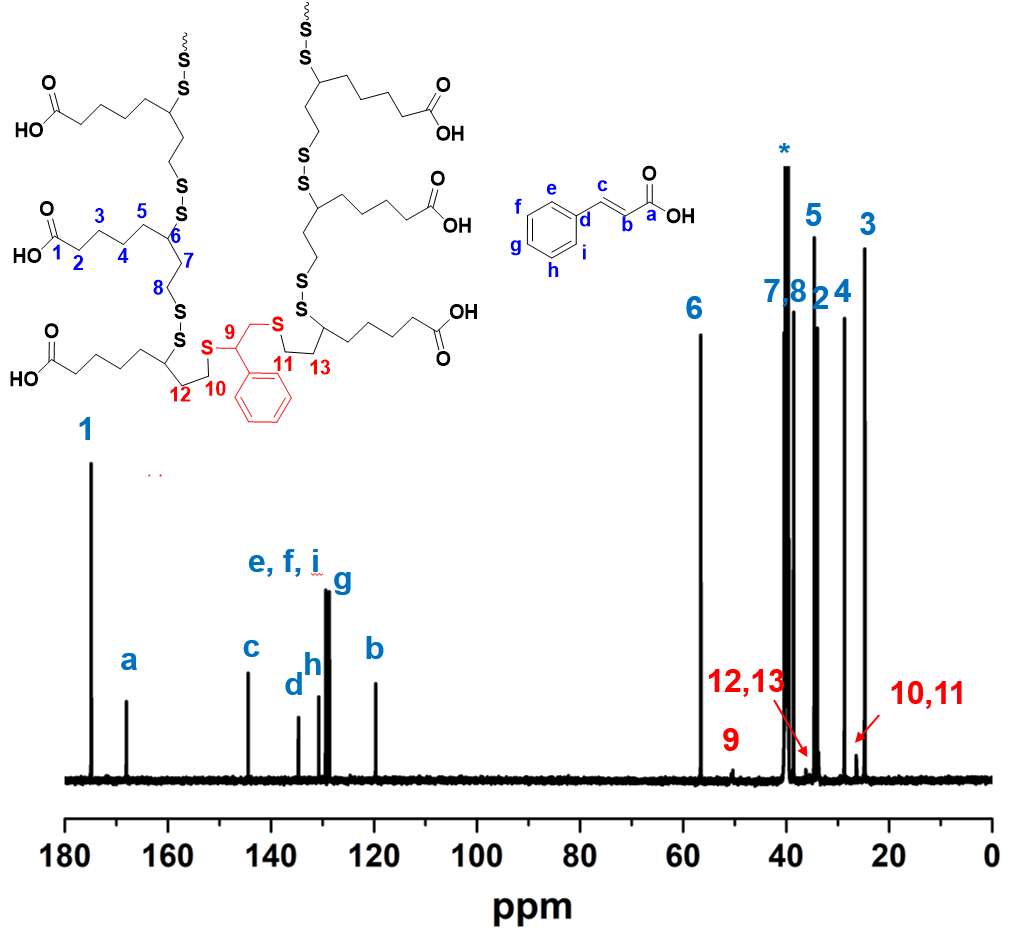


**Figure S6** ^13^C NMR spectrum of thioctic acid-cinnamic acid copolymer (CA@TA), the solvent was d_6_-DMSO.


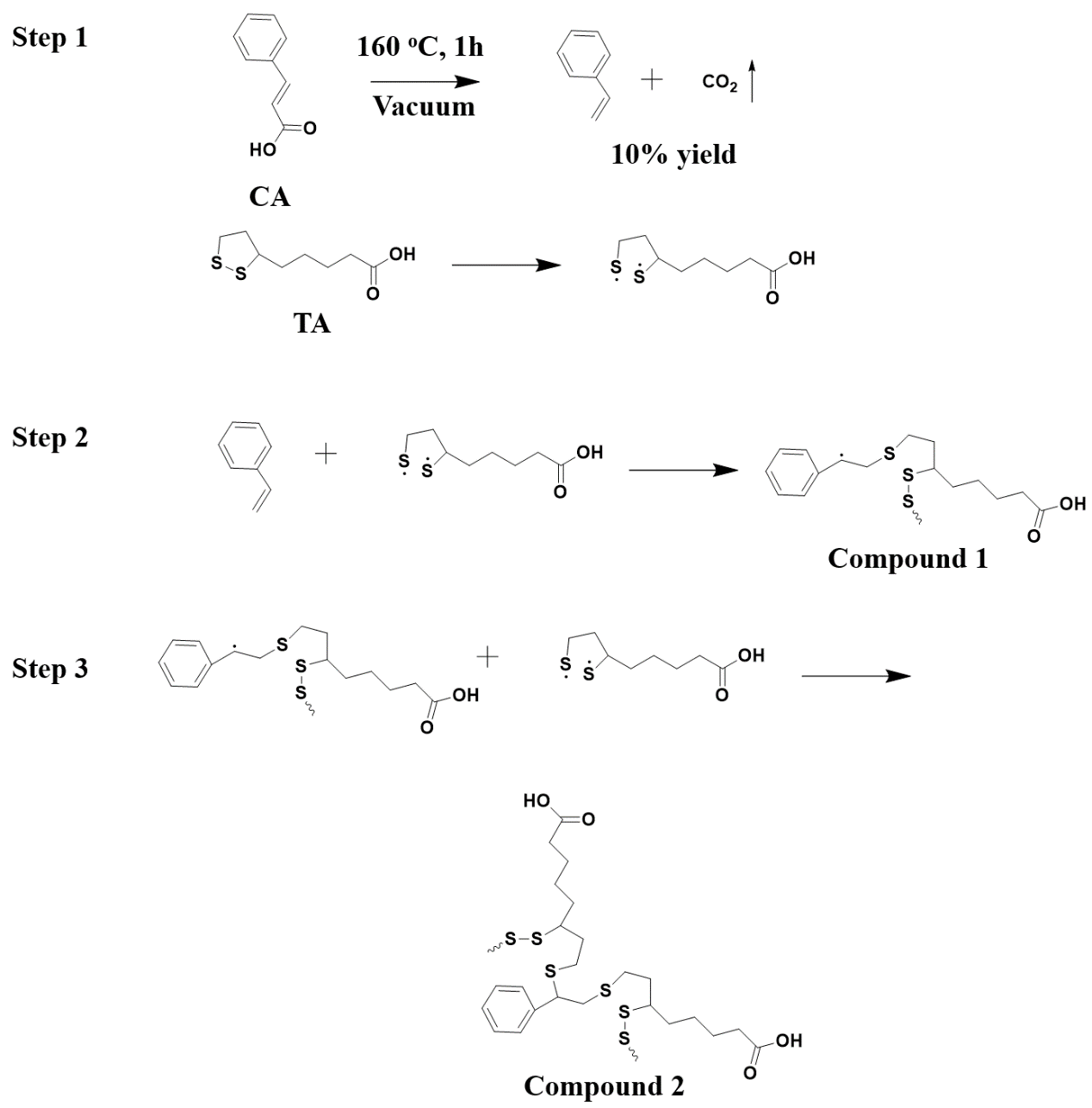


**Scheme S1** The mechanism of the reaction between CA and TA with a molar ratio of 1/6 at 160 ^º^C, -0.1 MPa for 1h.


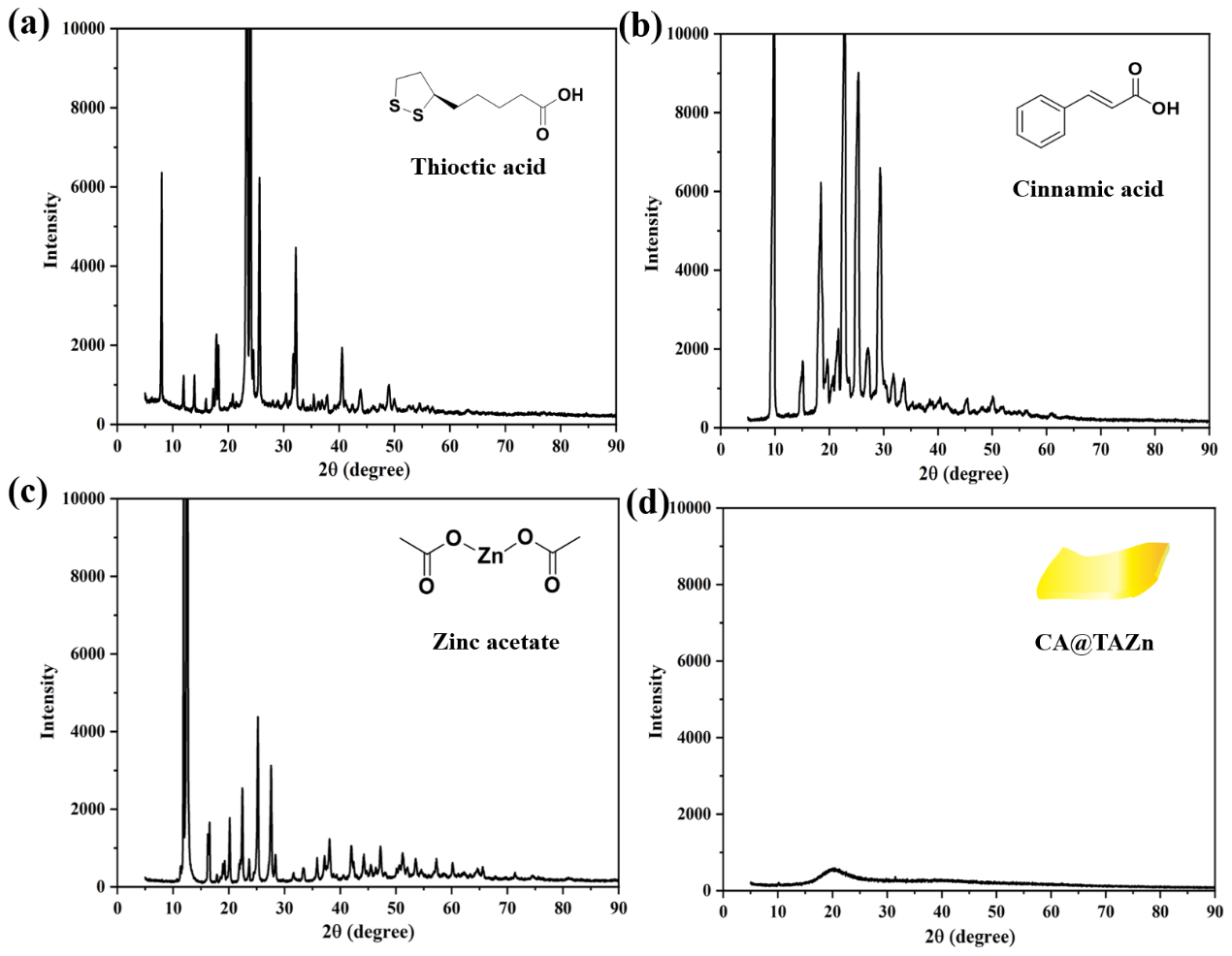


**Figure S7** XRD spectra of thioctic acid, cinnamic, zinc acetate dehydrate and CA@TAZn-1 gel patch.


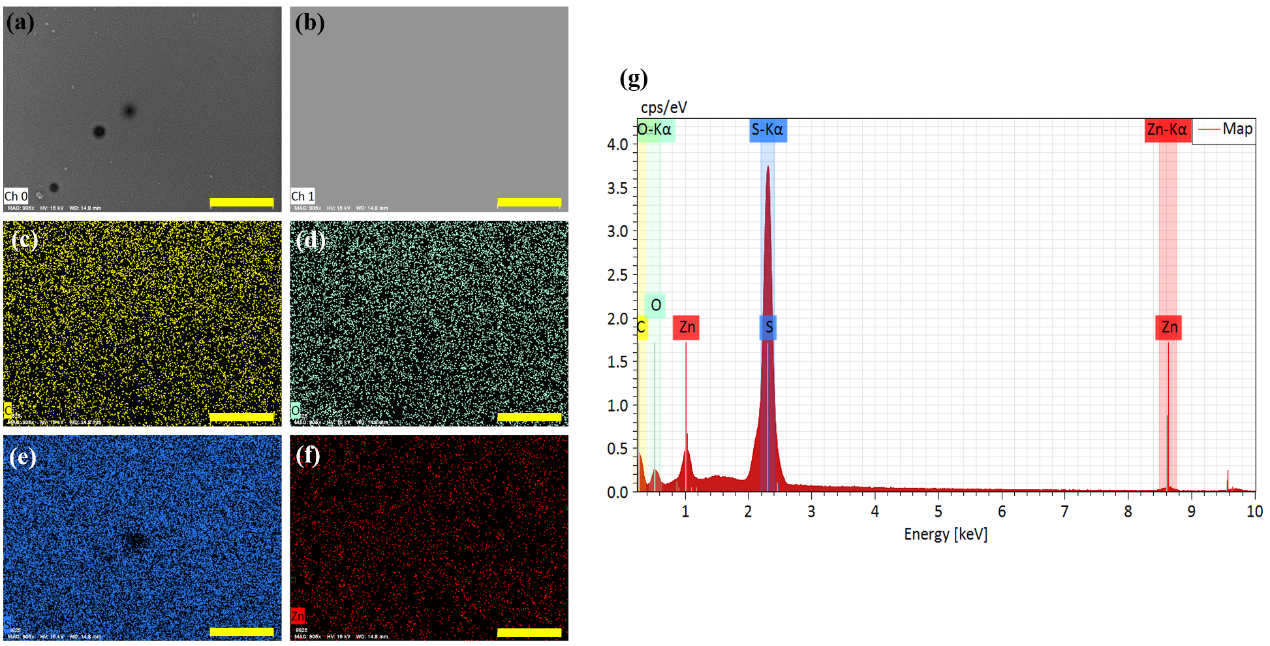


**Figure S8** SEM images of CA@TAZn-1 gel patch. (a,b) The morphology of gel patch viewed by SEM, the scale bar was 30 μm; (c-f) Elementary mapping images of carbon, oxygen. sulfur and zinc, respectively; (g) The corresponding energy-dispersive spectroscopy spectrum.


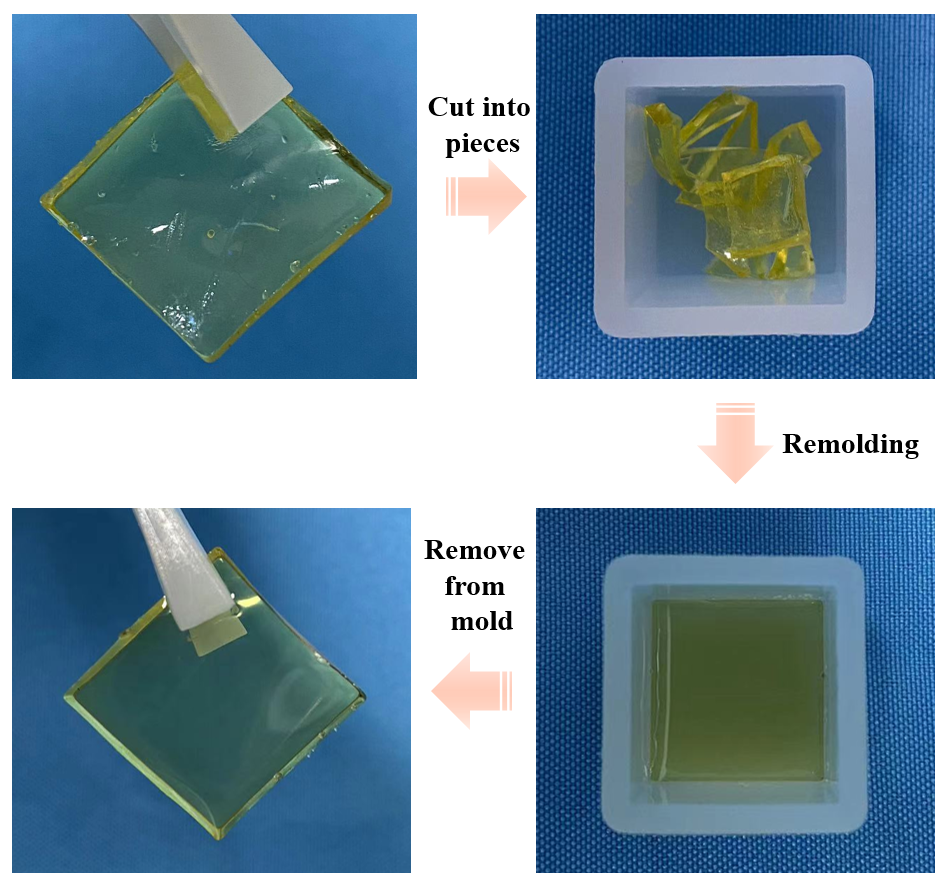


**Figure S9** The recyclability of CA@TAZn-1 gel patch.


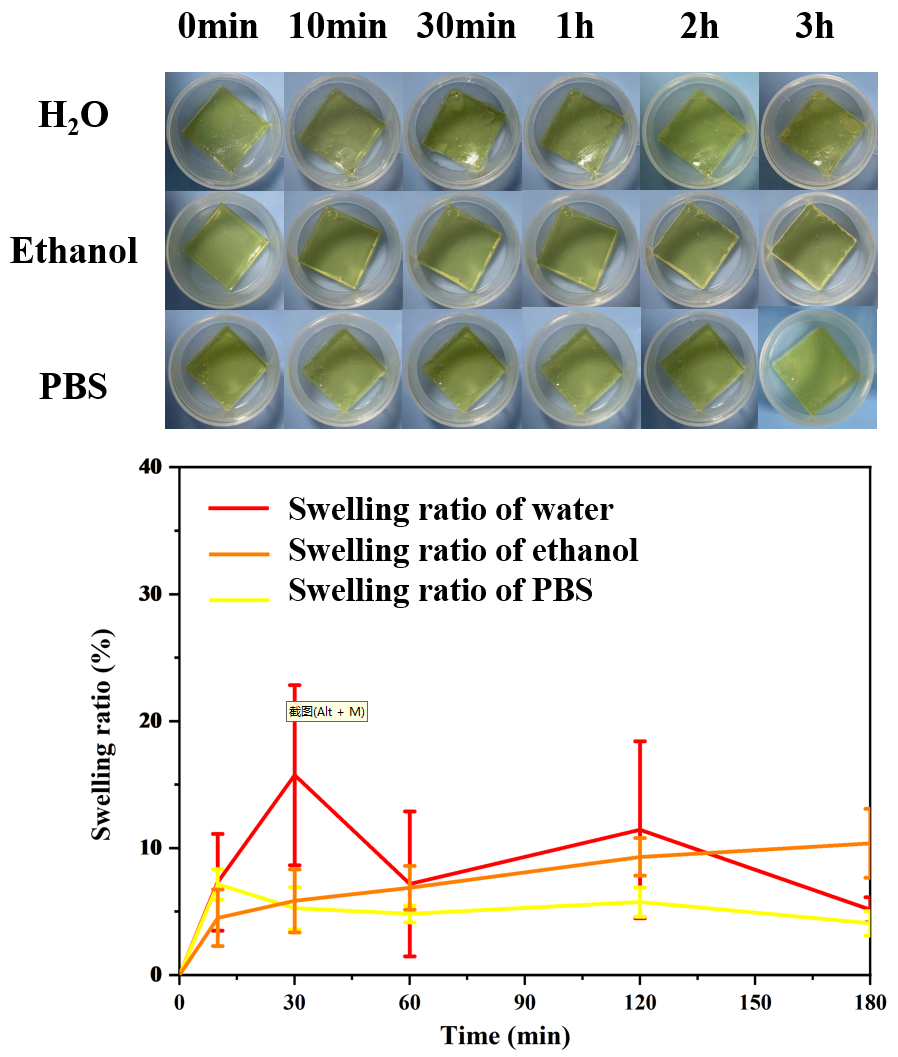


**Figure S10** Swelling ratios of CA@TAZn-1 gel patch in water, ethanol and PBS (n = 3).


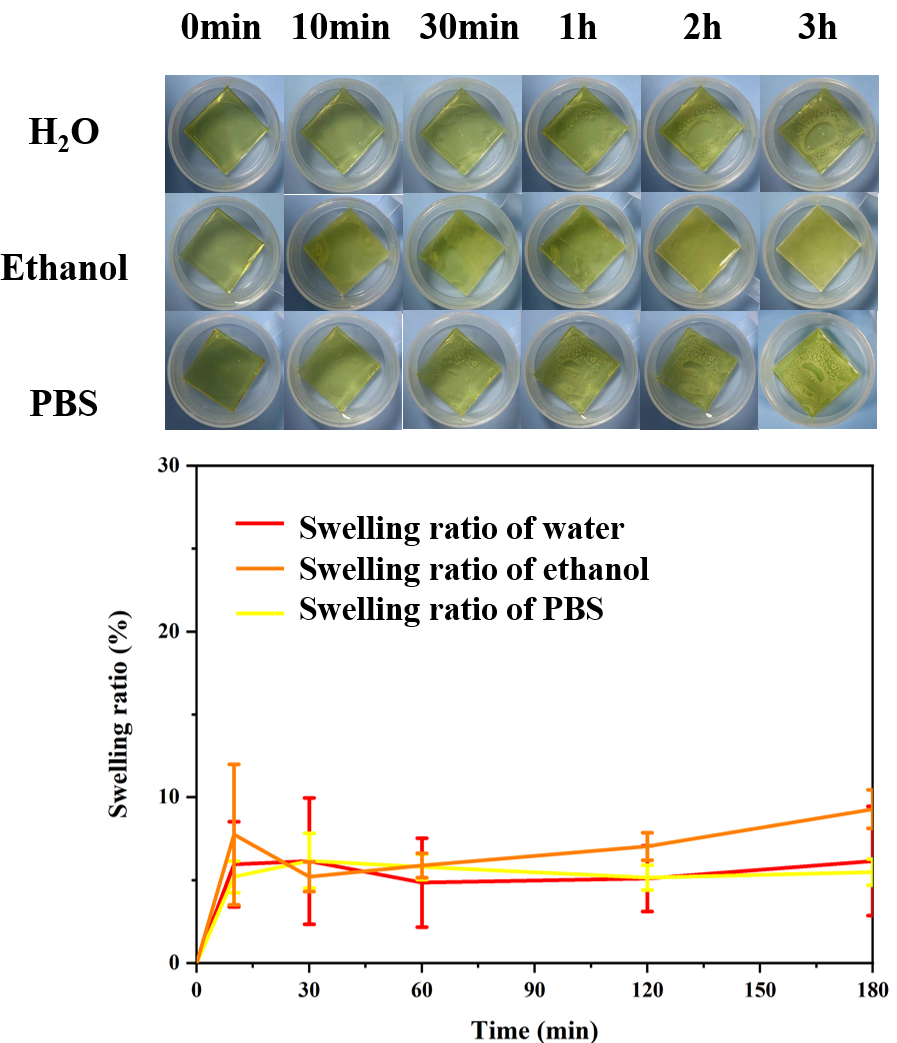


**Figure S11** Swelling ratios of CA@TAZn-2 gel patch in water, ethanol and PBS (n = 3).


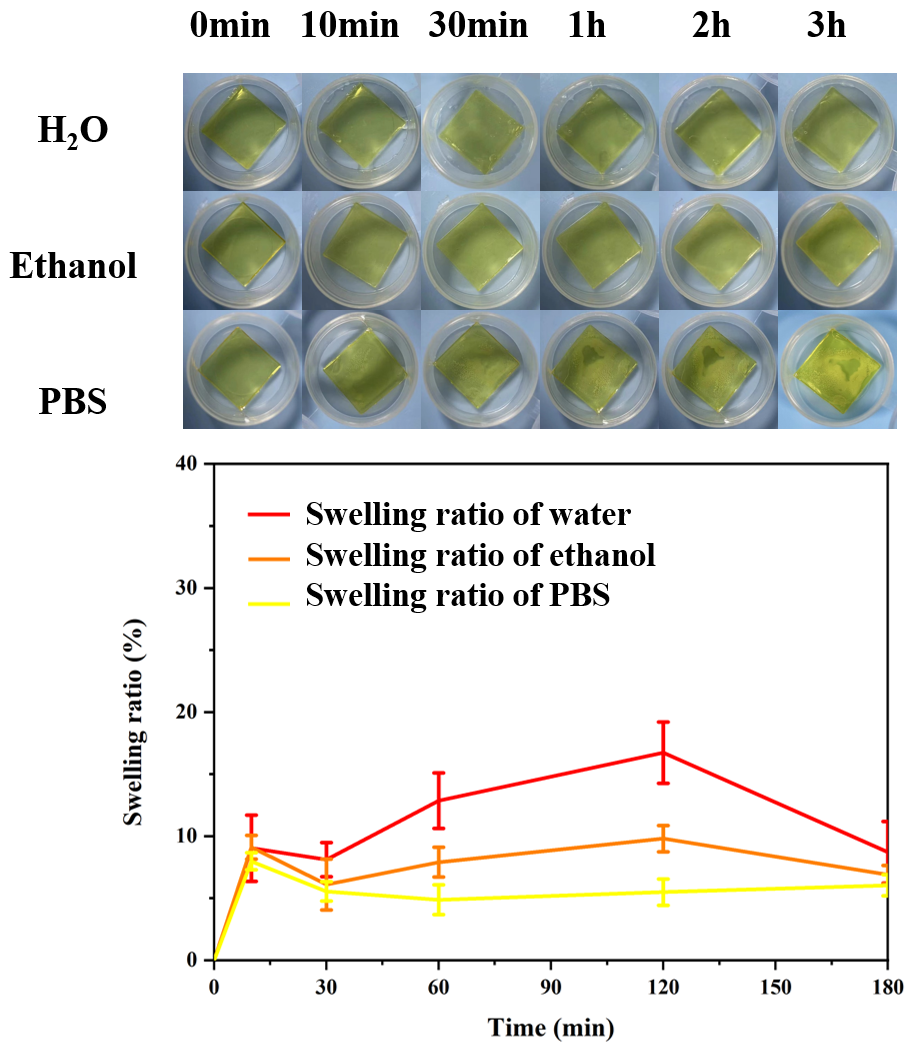


**Figure S12** Swelling ratios of CA@TAZn-3 gel patch in water, ethanol and PBS (n = 3).


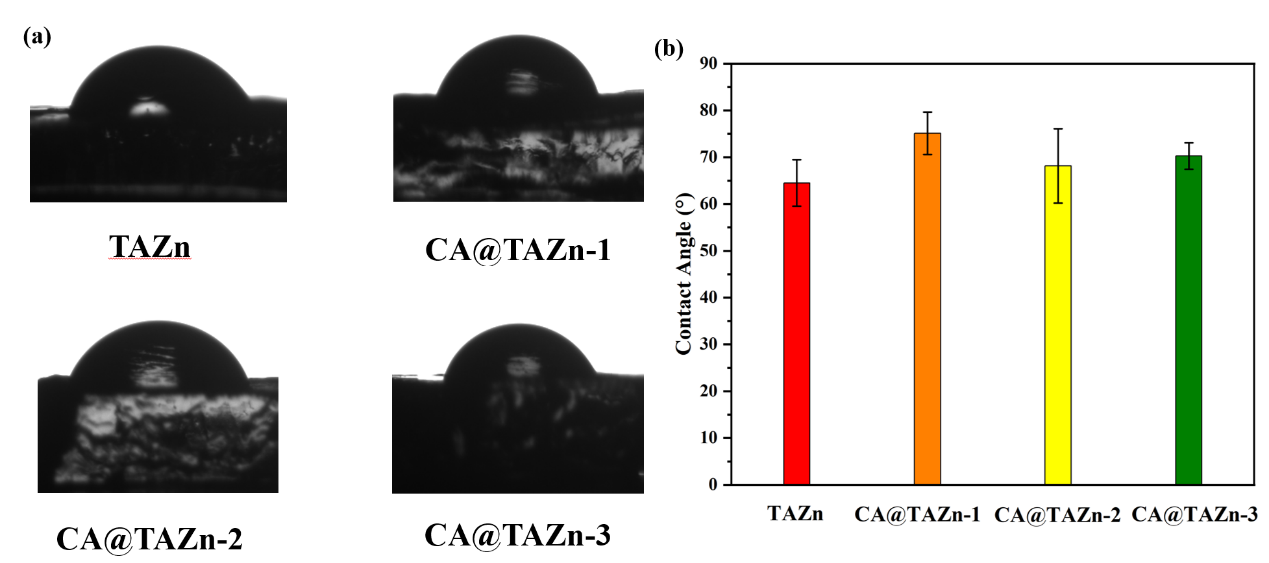


**Figure S13** (a) Image on a water droplet (2.0 μL) dipped onto the surface of TAZn, CA@TAZn-1, CA@TAZn-2, CA@TAZn-3 gel patches; (b) Water contact angles of TAZn, CA@TAZn-1, CA@TAZn-2, CA@TAZn-3 gel patches (n = 3).


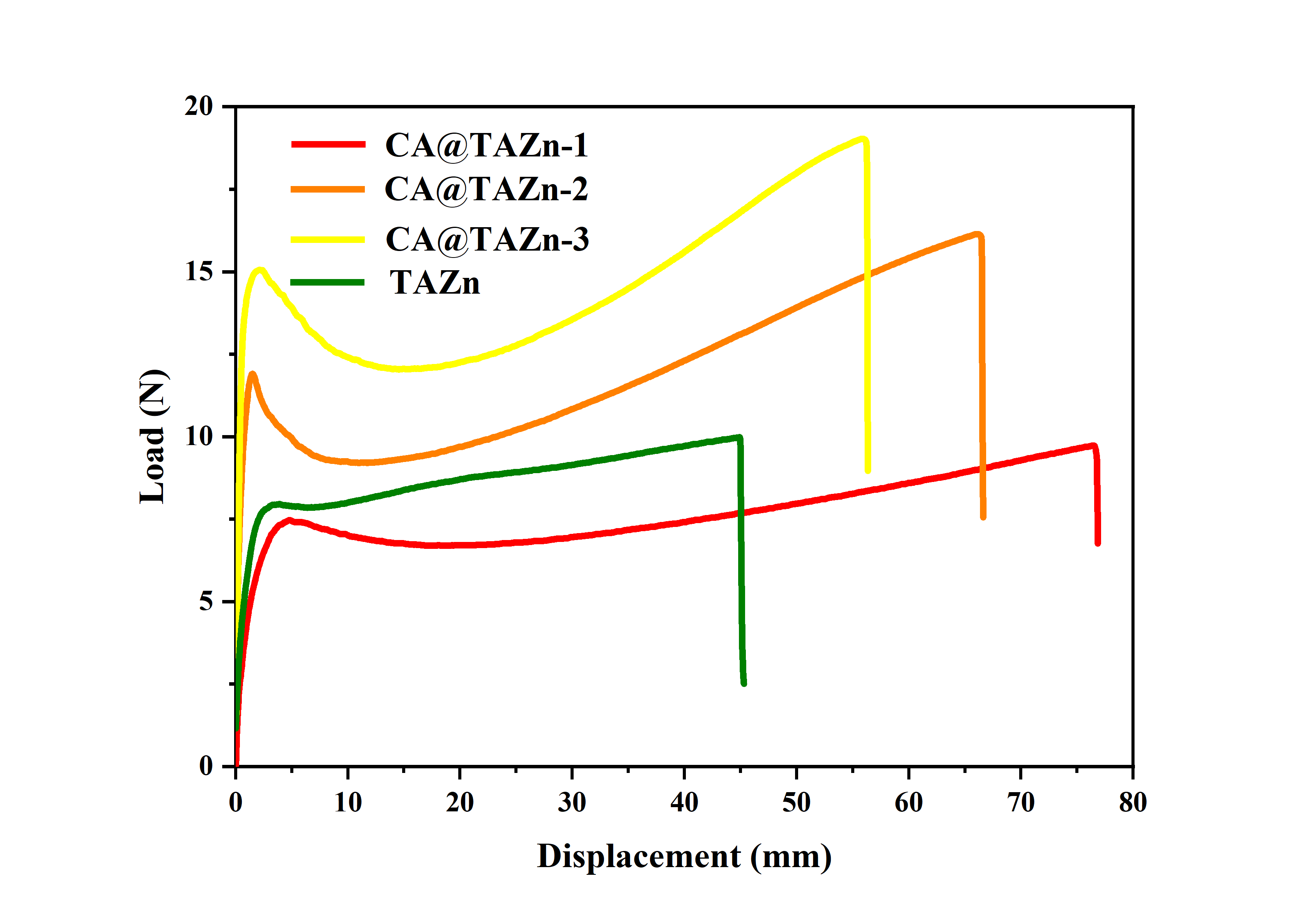


**Figure S14** Representative tensile curves of TAZn, CA@TAZn-1, CA@TAZn-2 and CA@TAZn-3 gel patches.


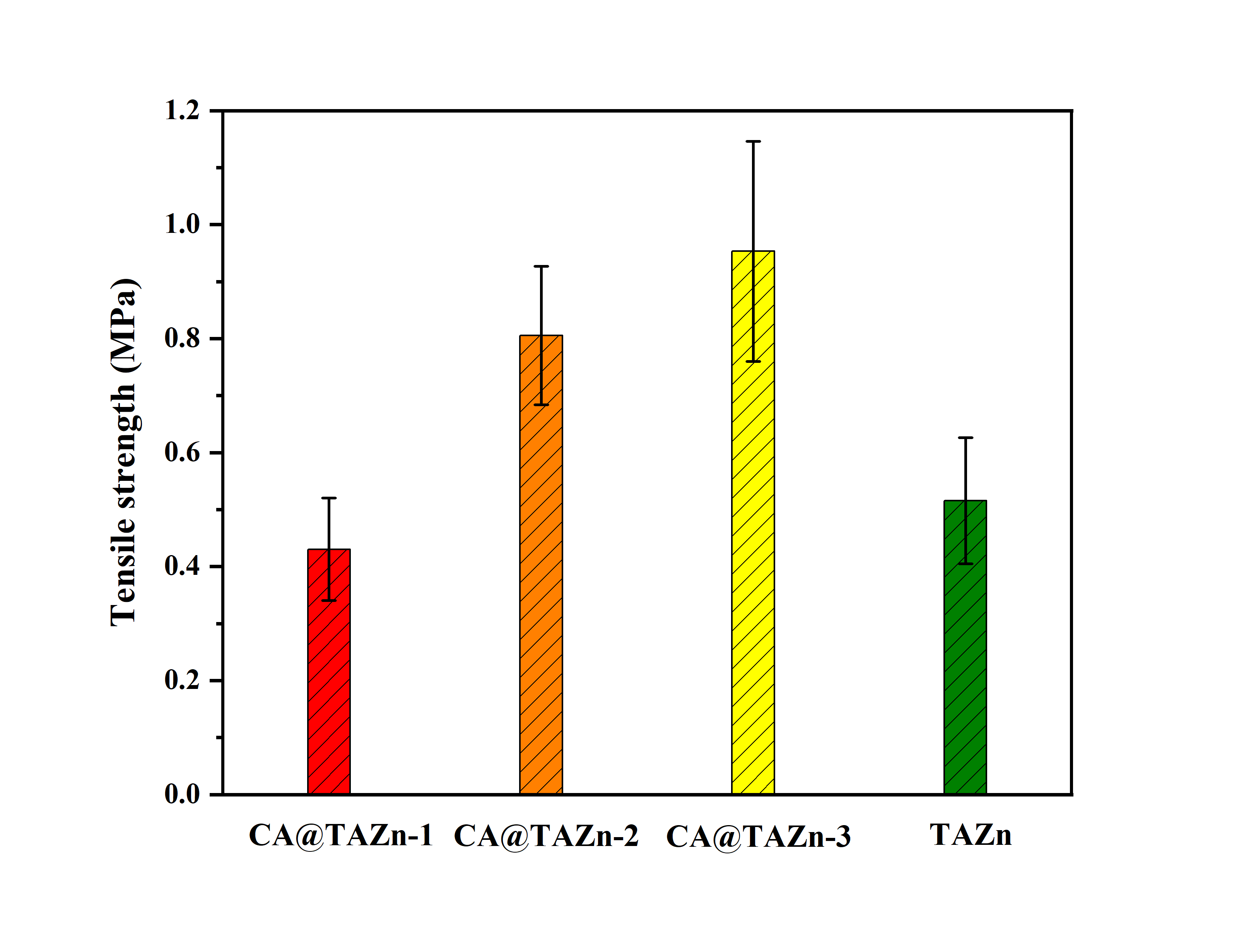


**Figure S15** The tensile strength of TAZn, CA@TAZn-1, CA@TAZn-2 and CA@TAZn-3 gel patches (n = 4).


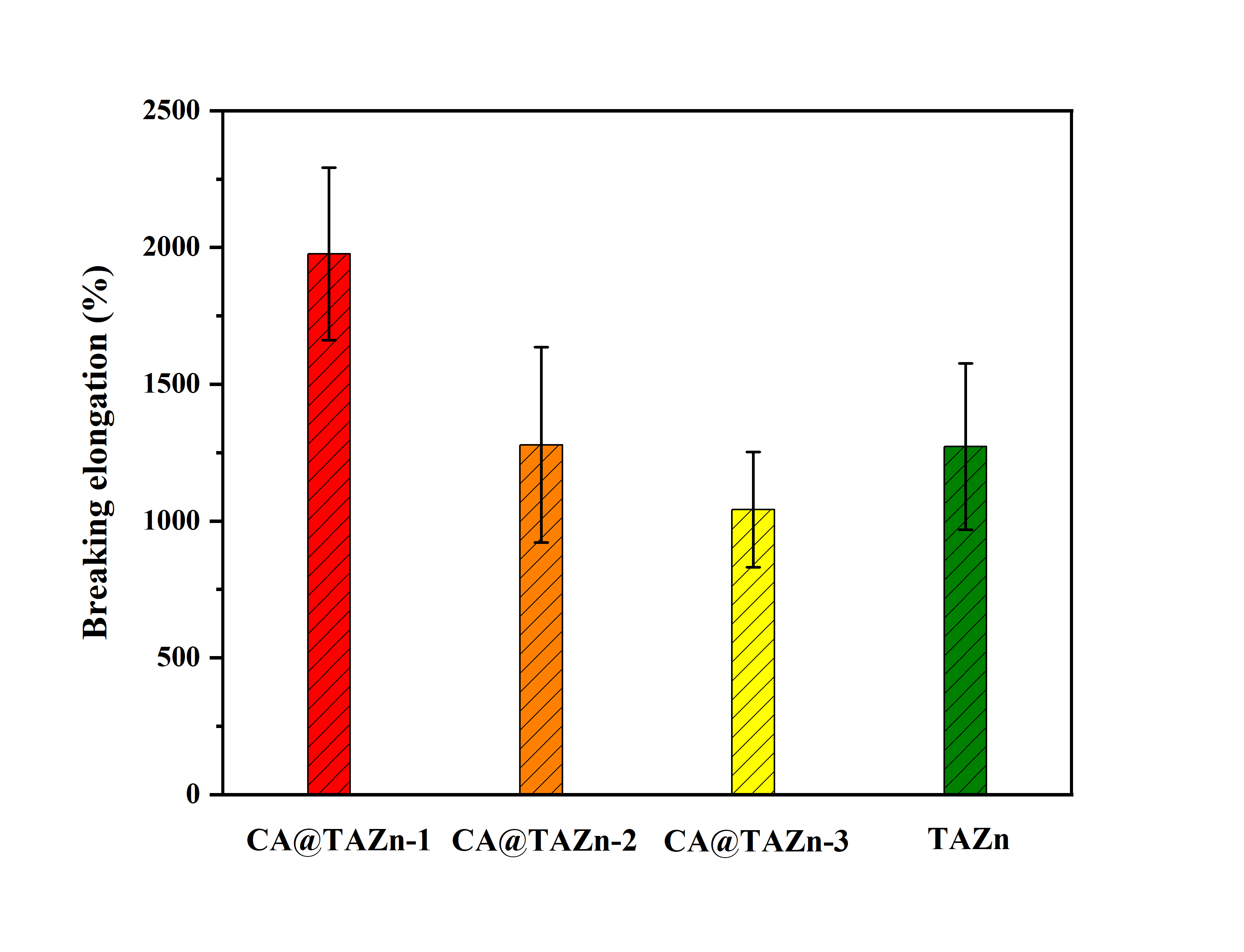


**Figure S16** The maximum breaking elongation of TAZn, CA@TAZn-1, CA@TAZn-2 and CA@TAZn-3 gel patches (n = 4).


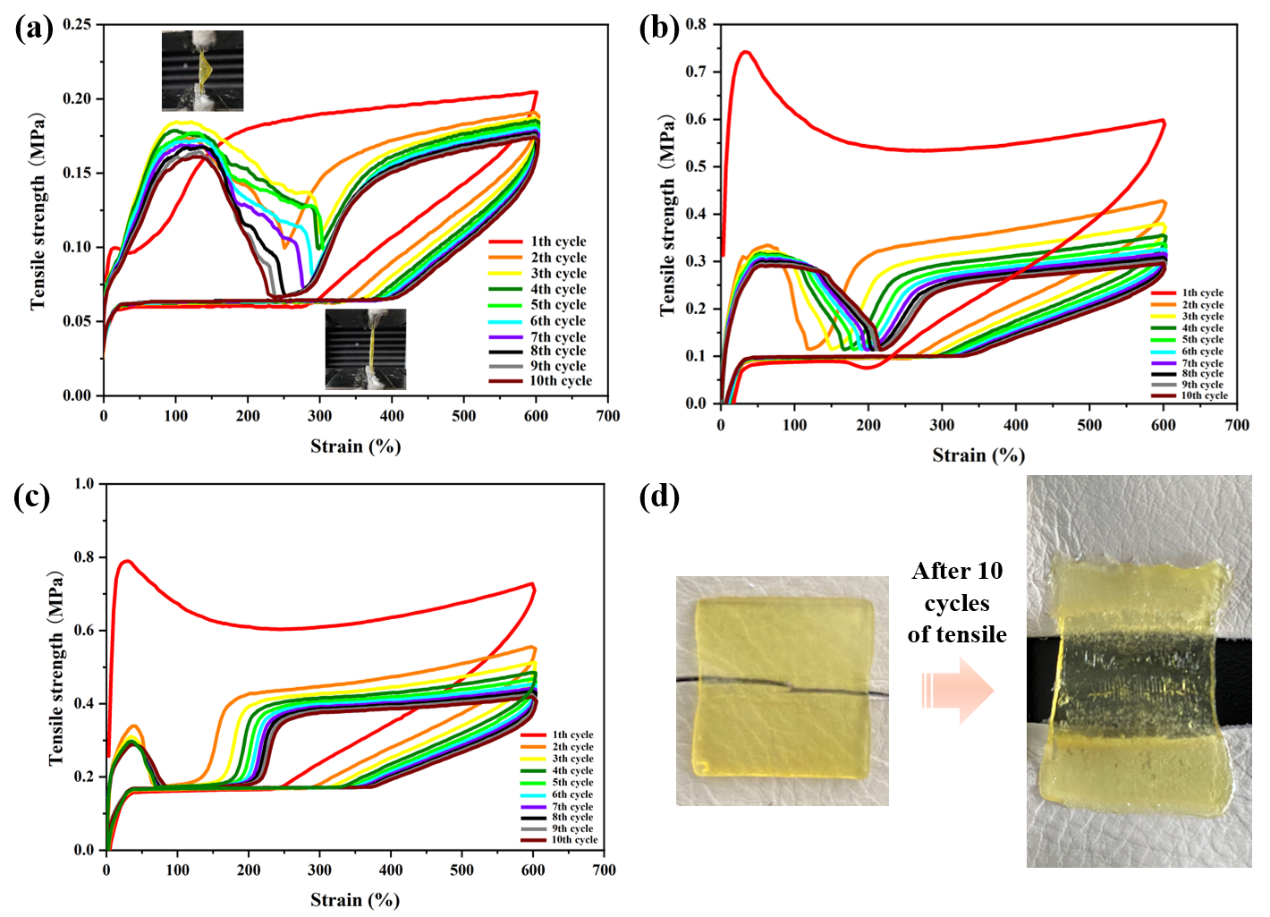


**Figure S17** (a-c) Cyclic strain-stress curves of CA@TAZn-1, CA@TAZn-2 and CA@TAZn-3 gel patches; (d) images on CA@TAZn-1 gel patches before and after 10 cycles of tensile.


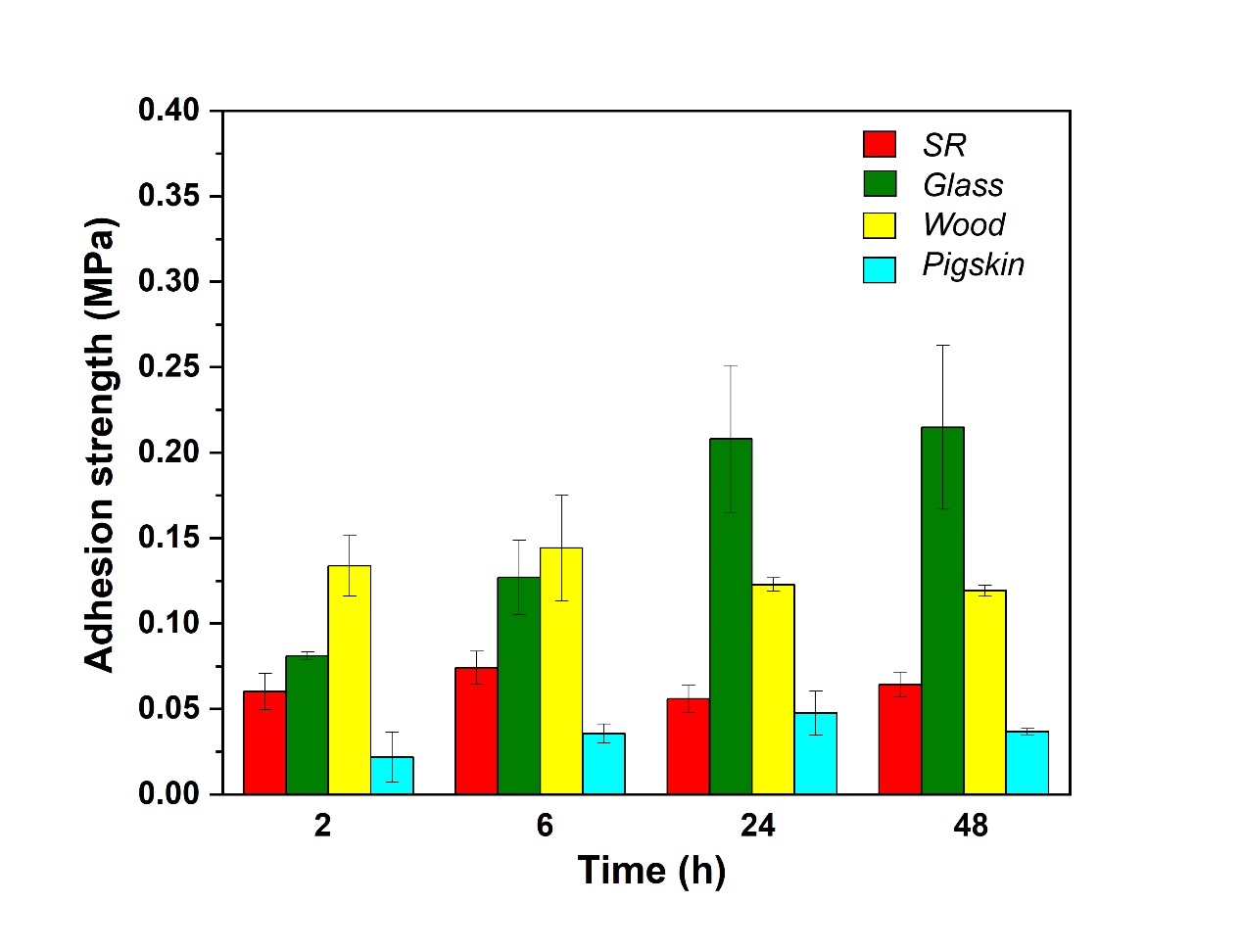


**Figure S18** Normal adhesion strength of CA@TAZn-1 gel patch with various materials at different adhesion periods.


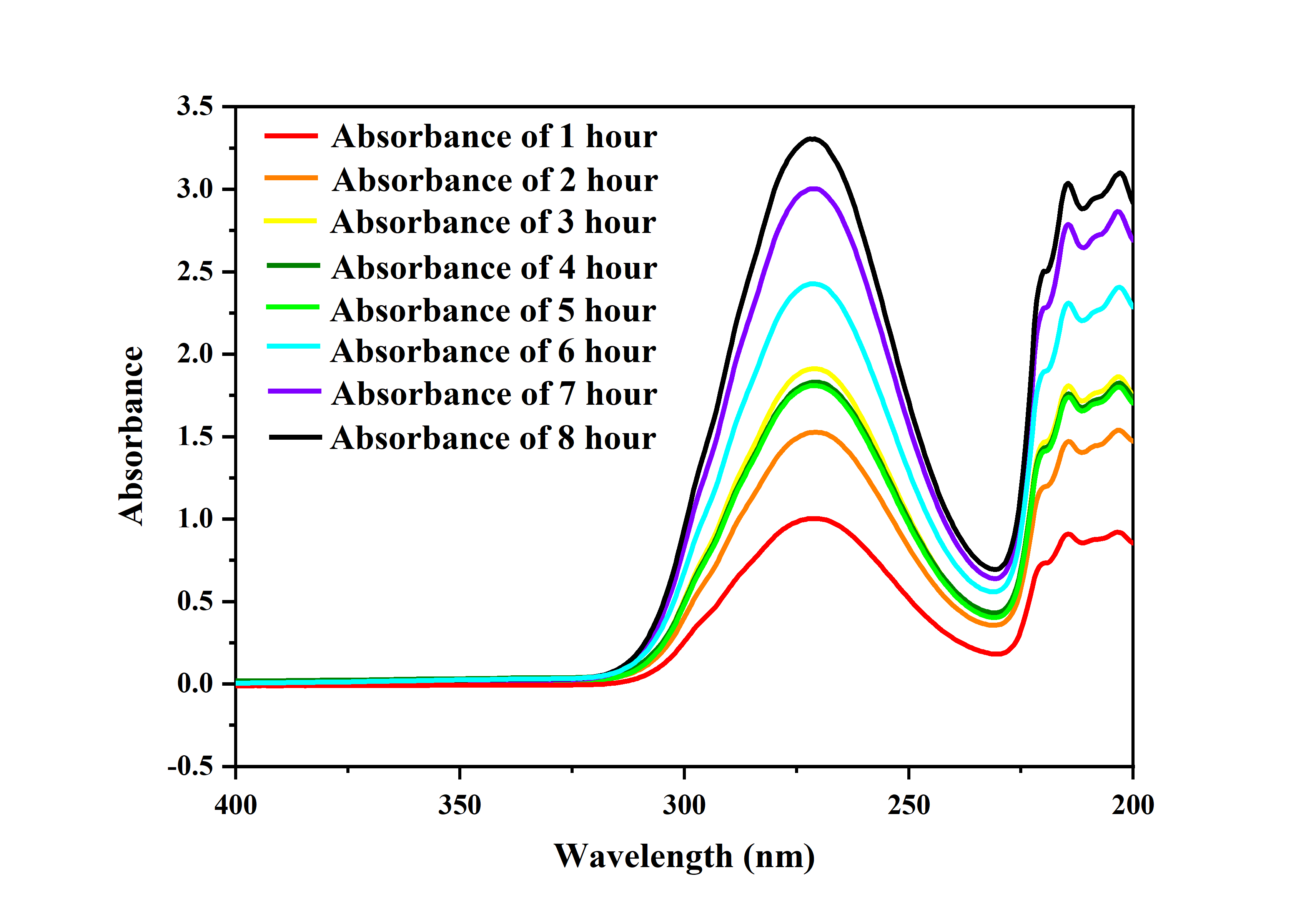


**Figure S19** The CA release behavior of CA@TAZn-1 gel patch (10 mm square and 1mm thickness) in 50 mL PBS solution viewed by UV-vis spectra.


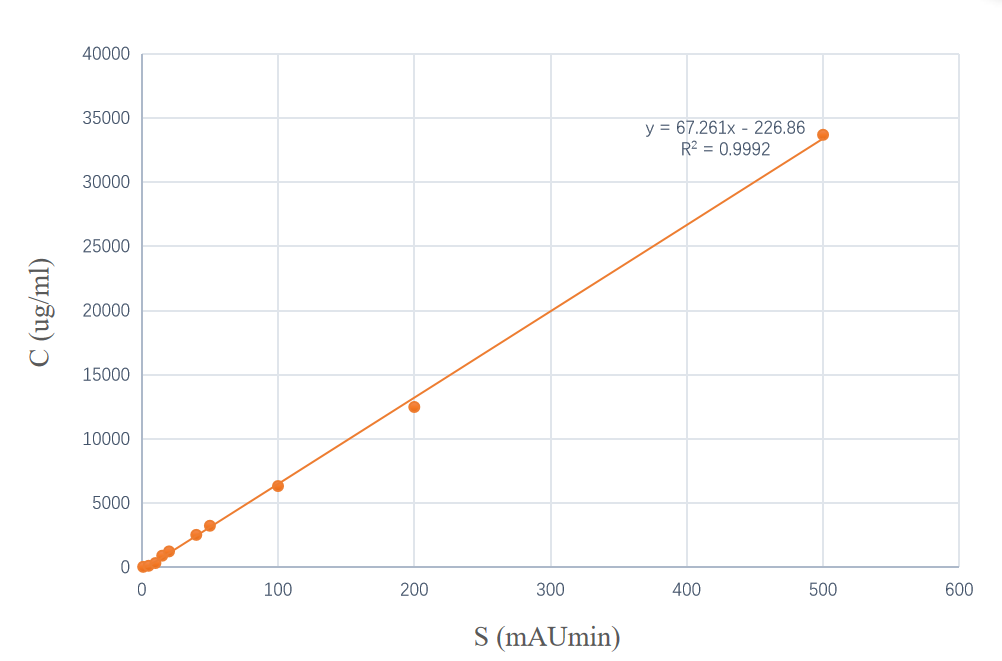


**Figure S20** The standard curve of cinnamic acid under a HPLC condition of 45 °C, 0.3% phosphoric acid solution/acetonitrile = 65/35, 1mL/min.


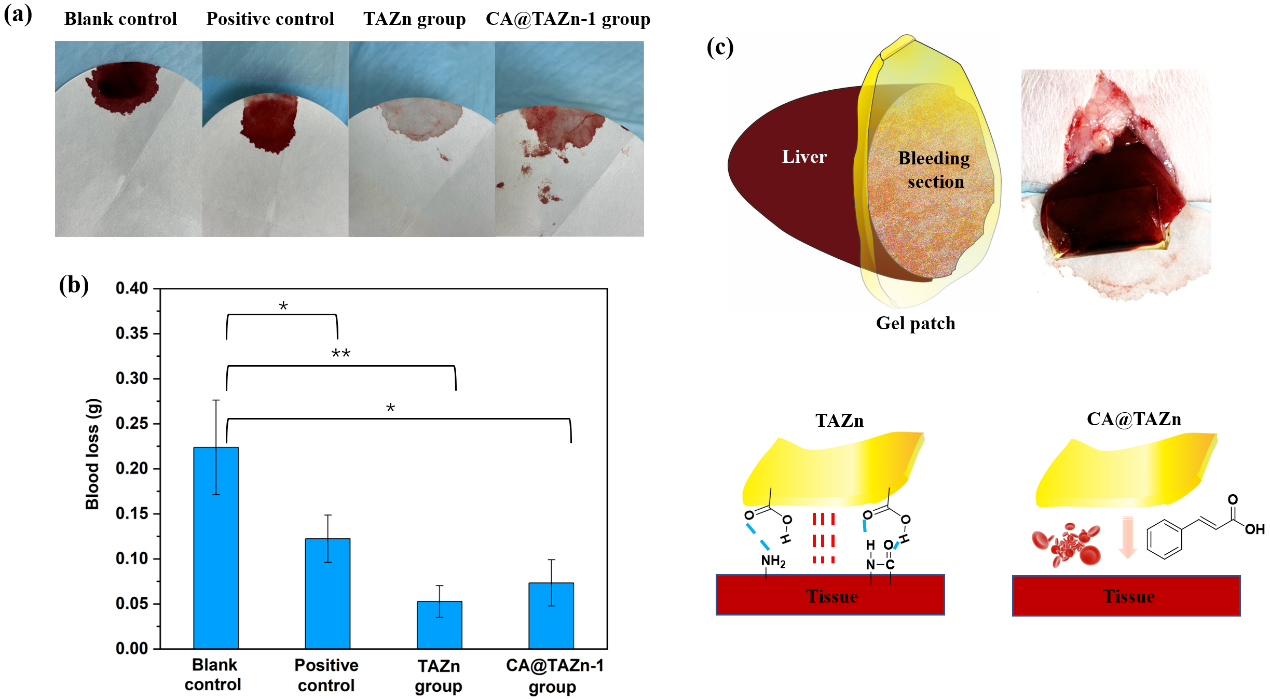


**Figure S21** The hemostatic effect of TAZn and CA@TAZn-1 gel patches in a liver hemostasis model of SD rats. (a) Images reflected on the injured liver bleeding treated with different methods; (b) The accumulated blood loss from liver bleeding of SD rats with different treatments ((n = 3, *P < 0.05, **P < 0.01); (c) Schematic illustration of the hemostatic effect of the related gel patches.

**Table S1** Comparison among different CA loading systems. ^a^The volume of liposome aqueous solution was unknown. ^b^This work does not contain quantitative study on the absorbed CA.

| Substrates | Method for solubilizing CA | Loading capacity | Assessment of the preparation method | References |
| --- | --- | --- | --- | --- |
| Transfersome | Emulsification by sodium deoxycholate and Lipoid S 100 | 1-2 mg/g | Advantage: Transfersomes can be used as carriers to enhance  the transdermal delivery of CA.  Drawbacks: (i) Organic solvent was used in the preparation procedure; (ii) Tedious time-consuming preparation procedure . | [S1] |
| Sea buckthorn seed oil gel | CA-sea buckthorn seed oil conjugate | 40-100 mg/g | Advantages: (i) Natural plant oil was used as the gel substrate; (ii) Wide range of gel-forming temperature; Drawbacks: (i) No *in vitro* CA release behavior testing; (iii) No *in vivo* testing aimed at practical uses. | [S2] |
| Liposome | Encapsulation by soya lecithin and cholesterol | -^a^ | Drawbacks: (i) Organic solvent was used in the preparation procedure; (ii) Limited loading capacity. (iii) Drug loss during encapsulation and the selection of liposome particle size. | [S3] |
| Polymer-based food packaging film | Sodium alginate-pectin incorporated with cinnamic acid | Approximately 90 mg/g | Advantages: (i) Natural derived polymer was used as the film substrate; (ii) Excellent degradability in soil; (iii) The film can be used for food preservation.  Drawback: No *in vivo* testing for biomedical uses though the film could gradually release CA. | [S4] |
| PLA film | CA was used as antimicrobial agents into PLA matrices | 10-20 mg/g | Advantage: Straightforward and robust synthetic route without solvent.  Drawback: No *in vivo* testing for biomedical uses though the film could gradually release CA. | [S5] |
| Zn-Al layered double hydroxide | Arrangement of CA in Layered double hydroxide through pi-pi interaction | -^b^ | Drawbacks: (i) Tedious preparation method; (ii) No *in vivo* testing aimed at biomedical uses. | [S6] |
| CA@TAZn gel | The melting TA-zinc acetate mixture as a temporary solvent | 100-113 mg/g | Advantages: (i) Low-cost raw materials; (ii) Solvent free; (iii) High loading capacity of CA; (iv) Straightforward and robust synthetic route.  Drawbacks: (i) Undesired reaction between CA and TA; (ii) The preparation method is not suitable for thermally unstable active pharmaceutical ingredients | This work |

**Supporting Movies**

**Movie S1:** The application of CA@TAZn-1 gel patch as a waterproof tape. The movie is real-time.

**Movie S2:** The underwater adhesion property of CA@TAZn-1 gel patch. The movie is real-time.

**Movie S3:** Hemostatic properties of TAZn gel patch. The movie is real-time.

**References**

[S1] Zhang YT, Xu YM, Zhang SJ, Zhao JH, Wang Z, Xu DQ, et al. In vivo microdialysis for the evaluation of transfersomes as a novel transdermal delivery vehicle for cinnamic acid. Drug Dev Ind Pharm, 2014;40:301-307. doi: https://doi.org/10.3109/03639045.2012.756888.

[S2] Gao YX, Li XQ, Yu YQ, Hou ZQ, Mu DH. Preparation and rheological properties of sea buckthorn seed oil gel. Food and Fermentation Industries 2020;56:119-127. doi: https://link.oversea.cnki.net/doi/10.13995/j.cnki.11-1802/ts.024188.

[S3] Parvathy PBS, Ravi RR, Sunil SR, Sharathkumar RM, Anand T. Biocompatible and sustained delivery of cinnamic acid using liposomal formulation. Res J Biotech 2024;19:9-21. doi: https://doi.org/10.25303/1905rjbt09021.

[S4] Tong WY, Rafee ARA, Leong CR, Tan WN, Dailin DJ, Almarhoon ZM, et al. Development of sodium alginate-pectin biodegradable active food packaging flm containing cinnamic acid. Chemosphere 2023;336:139212. doi: https://doi.org/10.1016/j.chemosphere.2023.139212.

[S5] Ordoñez R, Atarés L, Chiralt A. Effect of ferulic and cinnamic acids on the functional and antimicrobial properties in thermo-processed PLA films. Food Packaging Shelf 2022;33: 100882. doi: https://doi.org/10.1016/j.fpsl.2022.100882.

[S6] KimT, Paek, SM, Wang KK, Park JK, Salles F, Oh JM. Controlled molecular arrangement of cinnamic acid in layered double hydroxide through pi-pi interaction for controlled release. Int J Mol Sci 2024;25:4506. doi: https://doi.org/10.3390/ijms25084506.
